# Qualitative speed-accuracy tradeoff effects that cannot be explained by the diffusion model under the selective influence assumption

**DOI:** 10.1038/s41598-020-79765-2

**Published:** 2021-01-08

**Authors:** Farshad Rafiei, Dobromir Rahnev

**Affiliations:** School of Psychology, Georgia Institute of Technology, 654 Cherry Str NW, Atlanta, GA 30332 USA

**Keywords:** Human behaviour, Computational neuroscience

## Abstract

It is often thought that the diffusion model explains all effects related to the speed-accuracy tradeoff (SAT) but this has previously been examined with only a few SAT conditions or only a few subjects. Here we collected data from 20 subjects who performed a perceptual discrimination task with five different difficulty levels and five different SAT conditions (5000 trials/subject). We found that the five SAT conditions produced robustly U-shaped curves for (i) the difference between error and correct response times (RTs), (ii) the ratio of the standard deviation and mean of the RT distributions, and (iii) the skewness of the RT distributions. Critically, the diffusion model where only drift rate varies with contrast and only boundary varies with SAT could not account for any of the three U-shaped curves. Further, allowing all parameters to vary across conditions revealed that both the SAT and difficulty manipulations resulted in substantial modulations in every model parameter, while still providing imperfect fits to the data. These findings demonstrate that the diffusion model cannot fully explain the effects of SAT and establishes three robust but challenging effects that models of SAT should account for.

## Introduction

Humans have the ability to shorten the time it takes to decide at the expense of the decision’s accuracy, a phenomenon known as "speed-accuracy tradeoff" (SAT)^1^. SAT is a ubiquitous phenomenon that occurs in humans^2^, monkeys^3^, rodents^4^, and even insects^5^. Further, SAT is observed for virtually all types of decisions from pollinators choosing between flower species to simple perceptual judgments^6^. Understanding the mechanisms behind SAT has thus been a central goal of computational models for decades^7^.

**Figure 1:**
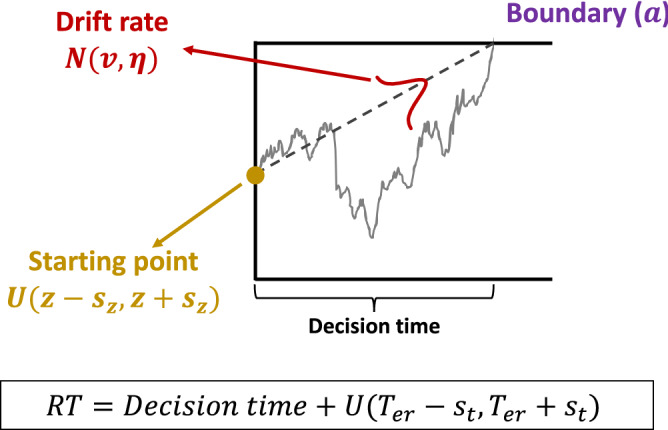
Schematic of the diffusion model. According to the diffusion model, perceptual decisions are the result of a noisy accumulation-to-bound process. An example trial of such a process is depicted. The standard diffusion model has seven free parameters: boundary ($a$), drift rate ($v$), non-decision time ($T_{er}$), starting point of the accumulation ($z$), as well as variability parameters for the drift rate ($\eta$), the non-decision time ($s_{t}$), and the starting point of the accumulation ($s_{z}$). The drift rate is assumed to have Gaussian variability across trials, whereas the starting point and non-decision time come from uniform distributions. Under a selective influence assumption in the diffusion model framework, SAT manipulations result in a change in the boundary parameter $a$ and difficulty manipulations result in a change in the drift rate parameter $v$.

The dominant way of conceptualizing of SAT is within the context of sequential sampling models^8^, and especially their most prominent member—the diffusion model^9,10^. The diffusion model conceptualizes the decision process as a noisy accumulation to a boundary. The boundary can be adjusted at will by the subject such that a low boundary would produce fast but inaccurate decisions, whereas a high boundary would produce slow but accurate decisions (Fig. 1). This mechanism provides a simple and intuitive way of accounting for SAT. In addition to the boundary (denoted by the parameter $a$), the diffusion model has parameters for drift rate ($v$), non-decision time ($T_{er}$), starting point of the accumulation ($z$), as well as variability parameters for the drift rate ($\eta$), the non-decision time ($s_{t}$), and the starting point of the accumulation ($s_{z}$).

Beyond simply accounting for the basic SAT effect of mean response time (RT) trading off against mean accuracy, the diffusion can reproduce many behavioral findings related to RT^8^. The model is thought to explain (i) the difference between the mean RT for error and correct trials, (ii) the relationship between the mean and standard deviation of RT distributions, and (iii) the shape of the RT distributions. We briefly review the research on each of these three areas below.

Explaining the difference between the mean RT for error and correct trials has been a driving force in the development of the diffusion model. Early models from the sequential sampling framework did not contain any variability parameters^11–13^. These models predicted that error and correct trials should produce the same mean RTs. However, depending on the condition, error RTs are either faster or slower than correct RTs^14^. To allow the model to explain these effects, two variability parameters were added. Specifically, drift rate variability was introduced to account for slow error trials^10^ and subsequently starting point variability was added to account for fast error trials^15^. (A third variability parameter—non-decision time variability—was later introduced to account for very fast RTs^16^). Therefore, given that two of its seven parameters can be directly traced to attempts to explain differences between mean RTs for error and correct trials, the full diffusion model should be expected to fit very well the pattern of RT differences between error and correct trials as a function of SAT.

The diffusion model has also been praised for explaining the relationship between the mean and standard deviation (SD) of the RT distribution. RT distributions with a low mean tend to have small SDs, whereas RT distributions with a high mean tend to have large SDs^17^. The diffusion model has been shown to make a strong prediction about the exact relationship between these two quantities: there should be a linear relationship between the mean and SD of the RT distributions^18^. In an influential paper, Wagenmakers and Brown^19^ examined a number of different experimental manipulations and concluded that the relationship between mean and SD of the RT distributions is indeed linear (we return to these results in the Discussion). However, Wagenmakers and Brown did not specifically examine the effects of SAT manipulations. Indeed, they examined ten different datasets but only one of them featured a SAT manipulation and that manipulation only included two levels of SAT (fast vs. accurate). Therefore, it remains unclear whether the same linear relationship between RT mean and SD would be observed if a wider range of SAT levels is examined.

Finally, the diffusion model is thought to explain the shape of the RT distributions. In general, RT distributions tend to have a characteristic shape: they extend further to the right than to the left^17^. Mathematically, this characteristic of a distribution is quantified using the distribution’s skewness, which is a measure of the asymmetry of the distribution about its mean (distributions with heavy right tails have positive skewness). The diffusion model is thought to fit precisely the shape of individual RT distributions^10^. For example, one paper examined true and certain fake RT distributions and showed that the diffusion model can account for the true ones but not for the fake ones^20^ (though, it should be noted that only one parameter was allowed to vary in those fits). Nevertheless, the skewness of RT distributions as a function of SAT has not been described and it is unclear whether the diffusion model can account for the change of skewness with SAT.

In the current study, we empirically tested how SAT affects the RT difference between error and correct trials, the relationship between the mean and SD of RT distributions, and the skewness of the RT distributions. We conducted a large study in which 20 subjects completed 5000 trials each. Each subject experienced five different SAT settings spanning from extreme speed pressure (designed to produce performance close to chance) to no speed pressure at all. In addition, we used five different levels of contrast that allowed us to examine whether the patterns of results depend on task difficulty. To anticipate, we found that SAT led to U-shaped functions for the mean RT difference between error and correct trials, the ratio of mean to SD of the RT distribution, and the skewness of the RT distribution. Critically, the diffusion model where only drift rate varies with contrast and only boundary varies with SAT could not account for any of these results. Further, allowing all parameters to vary across conditions showed violations of the “selective influence” assumption^21^—the notion that SAT and stimulus contrast should only affect the boundary and drift rate, respectively—for every single model parameter. These results demonstrate that the current version of the diffusion model cannot fully account for the effects of SAT on behavior.

## Results

**Figure 2:**
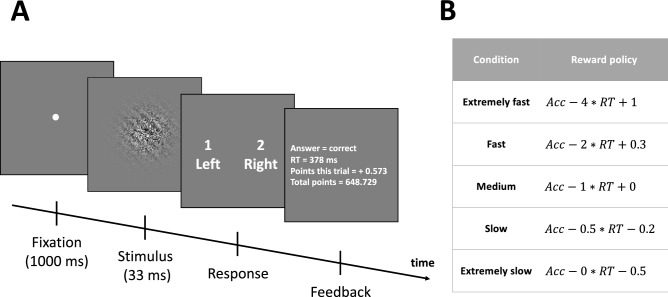
Trial sequence. (**A**) An example trial. Subjects indicated whether a Gabor patch was oriented counterclockwise (“left”) or clockwise (“right”) from vertical. The Gabor patch was presented for 33 ms and was preceded by a fixation period of 1000 ms. After indicating their response, subjects received detailed feedback on their performance. (**B**) The experiment included five different speed-accuracy tradeoff conditions—“extremely fast,” “fast,” “medium,” “slow,” and “extremely slow.” Each condition was blocked and featured a different penalty on response time (RT) computed in seconds. Acc, accuracy on the current trial (1 for correct responses, 0 for wrong responses).

We collected a large sample of 20 subjects each completing 5000 trials over five different sessions. Subjects performed a simple perceptual task that required discriminating between clockwise and counterclockwise orientations of a briefly presented (33 ms) Gabor patch (Fig. 2). Each subject experienced five SAT levels and five Gabor contrasts creating 25 independent conditions.

### Behavioral results

Five subjects were unable to provide sufficiently fast decisions in the “extremely fast” SAT condition (individual data are plotted in Supplementary Fig. 1). These five subjects had the five highest average RTs in the “extremely fast” condition (mean across the five contrast levels = 378 ms), as well as the five highest d’ values (mean d’ across the five contrast values = 1.04). These values were considerably higher than the rest of the subjects (mean RT across contrasts = 277 ms; mean d′ across contrasts = 0.15). In fact, these five subjects exhibited performance in the “extremely fast” condition that was in line with the “fast” condition for the rest of the subjects (mean RT across contrasts = 384 ms, mean d′ across contrasts = 0.93). Therefore, in order to keep the conditions as different as possible between subjects, we excluded these five subjects from further analyses. However, we repeated all analyses from the rest of the paper without any exclusions and found very similar results (Supplementary Fig. 2).

Several of the qualitative results discussed below are driven by the “extremely fast” condition. One potential concern is that subjects may treat that condition as a purely detection task and thus perform in a qualitatively different fashion. However, even after the exclusions, the remaining subjects performed consistently above chance in the “extremely fast” condition (t(14) = 3.80, p = 0.002, Cohen's d = 0.98), thus demonstrating that they did not treat this condition as a pure detection task. Moreover, if the “extremely fast” condition is indeed qualitatively different, one would expect the (d’,RT) pairs on that condition to form a separate cluster, while the same pairs for the rest of the conditions should lie on a single continuum. However, plots of each individual’s (d′, RT) pairs demonstrated the existence of a single continuum along all conditions (Supplementary Fig. 3). Therefore, it does not appear that the “extremely fast” condition is qualitatively different from the remaining conditions. Consequently, we analyze all SAT conditions together but, for completeness, we also examine diffusion model fits that ignore the “extremely fast” condition.

#### *d*′*-RT curves*

**Figure 3:**
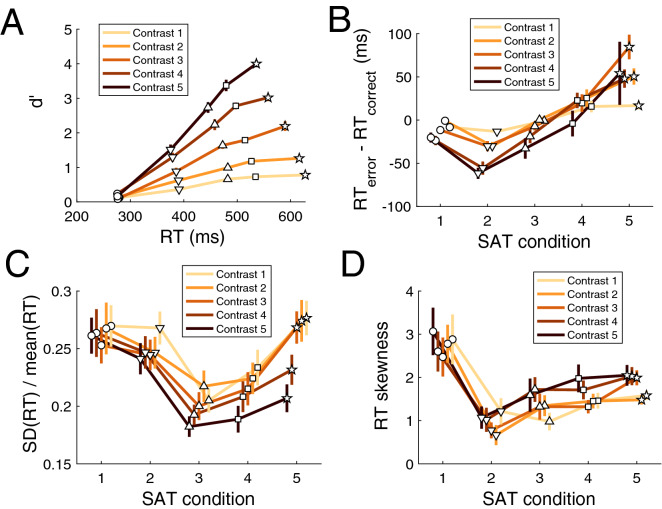
Effects of SAT. (**A**) The effect of SAT and contrast manipulations on d’ and mean RT. Higher contrasts resulted in higher d′ and lower RT, whereas stronger accuracy stress led to higher d′ and higher RT. All d′-RT curves are approximately linear. (**B**) RT difference between error and correct trials. The RT difference formed a robustly U-shaped curve as a function of SAT such that the minimum value (where error RTs are faster than correct RTs) occurred for the “fast” condition. On the other hand, the lack of any speed stress in the “extremely slow” condition resulted in error RTs being slower than correct RTs. (**C**) Ratio between the SD and mean of RT distributions as a function of SAT. The $\frac{{SD\left( {RT} \right)}}{{mean\left( {RT} \right)}}$ ratio exhibited a U-shaped curve as a function of SAT for all contrasts. In addition, the ratio monotonically decreased with higher contrasts. (D) RT distribution skewness. The skewness of the RT distribution formed robustly U-shaped curves for all contrasts with the minimum skewness occurring for the “fast” condition. In all subplots, lines represent different contrast levels, symbols represent different SAT conditions (circle: “extremely fast” condition; triangle pointing down: “fast” condition; triangle pointing up: “medium” condition; square: “slow” condition; star: “extremely slow” condition), and error bars represent S.E.M.

To examine the effects of SAT on performance, we first plotted the mean RT against d’ for each contrast and SAT condition (Fig. 3) and performed repeated measures ANOVAs with factors SAT and contrast. We found that, not surprisingly, higher contrasts resulted in lower RT (F(4,56) = 100.7, *p* = 6.8 × 10^–25^) and higher d′ (F(4,56) = 415.4, *p* = 6.6 × 10^–41^), whereas higher speed stress resulted in both lower RT (F(4,56) = 206.03, *p* = 8.7 × 10^–33^) and lower d′ (F(4,56) = 233.97, *p* = 3.1 × 10^–34^). Thus, our SAT manipulation and contrast levels were effective in altering subjects’ speed focus and the difficulty of the task, respectively.

As can be appreciated from Fig. 3A, the “extremely fast” condition produced performance very close to chance level with mean RT between 250 and 300 ms. Further, the increase in d’ for SAT conditions with a progressively higher emphasis on accuracy was roughly linear with mean RT. We tested for linearity by fitting a quadratic model $y = ax^{2} + bx + c$ to the data from each contrast of each subject and found that the average quadratic parameter *a* across the five contrasts was not significantly different from zero (*t*(14) = − 1.18, *p* = 0.26). Further, the same parameter was not significantly different from zero for any of the five contrasts individually (all *p*’s > 0.05). These results suggest that, at least in our specific task, SAT induces a roughly linear relationship between d′ and mean RT within the range of tested SAT conditions. (Note that it is possible to add SAT conditions that force subjects to be extra slow thus “extending” the d′-RT curve to the right. Such conditions will likely produce minimal gains in d’ but substantial increases in mean RT, thus breaking the linear relationship between d′ and mean RT).

#### RT difference between error and correct trials

Having established the success of our manipulations, we turned to quantifying how SAT influences the difference in mean RT between error and correct trials, which has been a driving force in the development of the diffusion model. We first examined whether the RT difference between error and correct mean RTs is sensitive to SAT and contrast. Indeed, a repeated measures ANOVA revealed both a main effect of SAT (F(4,56) = 28.2, *p* = 7.4 × 10^–13^) and a main effect of contrast (F(4,56) = 5.74, *p* = 0.0006), as well as a significant interaction between the two (F(14,48.9) = 1.86, *p* = 0.0004) (Fig. 3B). Critically, the RT difference appeared to show a robustly concave shape as a function of SAT. We quantified this effect by fitting a quadratic function to the data from each subject and each contrast level. We found that the quadratic coefficient averaged across contrasts was significantly positive (*t*(14) = 5.46, *p* = 0.00008), which indicates the presence of robustly U-shaped curves. When each contrast was examined separately, only contrast 1 did not produce a significantly U-shaped function (*t*(14) = 1.55, *p* = 0.14), whereas contrasts 2 through 5 each produced significantly U-shaped curves (*p* = 0.00001, 0.00001, 0.0001 and 0.026, respectively).

The overall U-shaped curve for the RT difference between error and correct trials appears to be the result of three different effects. First, the difference between error and correct RTs starts close to zero for the “extremely fast” condition (SAT condition 1 in Fig. 3B). There is a simple explanation for this effect: Performance in the “extremely fast” condition is close to chance and in such a regime there cannot be any differences between error and correct trials. Indeed, even though the average difference in RT for the “extremely fast” condition (− 12.9 ms) was significantly negative (t(14) = − 3.2, *p* = 0.006), this effect was driven by subjects who had above-chance performance as revealed by a strongly negative correlation between d′ and RT difference for the “extremely fast” condition (r = − 0.83, *p* = 0.0001). Second, the difference between error and correct RTs decreases as speed stress relaxes and performance becomes higher than chance in the “fast” condition (see Fig. 3A). Indeed, in our dataset the RT difference reaches its minimum for the “fast” condition (average difference = − 38 ms, t(14) = − 8.85, *p* = 4.2 × 10^–7^). Third, as the speed stress is further relaxed, there is a monotonic increase in the difference between error and correct RTs until it becomes significantly positive for the extremely slow condition (average difference = 51 ms, t(14) = 4.34, *p* = 0.0007). We speculate about the reasons for these effects in the “Discussion”.

#### Ratio between the standard deviation and mean of RT distributions

Beyond its ability to explain the difference between error and correct RTs, the diffusion model has been praised for its ability to predict a linear relationship between the mean and SD of RT distributions^8^. However, no previous paper has examined how SAT affects the SD-mean relationship. If the relationship between SD and mean of RT distributions is indeed linear such that $SD\left( {RT} \right) = a + b \times mean\left( {RT} \right)$, then the ratio between the SD and mean of the RT distribution, $\frac{{SD\left( {RT} \right)}}{{mean\left( {RT} \right)}}$, would equal $b + \frac{a}{{mean\left( {RT} \right)}}$. Therefore, this ratio should be either a monotonically increasing or a monotonically decreasing function of mean RT depending on the sign of the parameter $a$ (positive/negative values of $a$ would make the function monotonically decreasing/increasing).

Here we tested whether such monotonic function is observed when SAT is varied. We first performed a repeated measures ANOVA on the $\frac{{SD\left( {RT} \right)}}{{mean\left( {RT} \right)}}$ ratio with factors SAT and contrast. We found a main effect of SAT (F(4,56) = 7.6, *p* = 5.7 × 10^–5^), a main effect of contrast (F(4,56) = 16.89, *p* = 3.9 × 10^–9^), and an interaction between SAT and contrast (F(14,56.8) = 4.75, *p* = 6.7 × 10^–5^) (Fig. 3C). Generally, the $\frac{{SD\left( {RT} \right)}}{{mean\left( {RT} \right)}}$ ratio increased as contrast decreased with the lowest contrast giving rise to significantly higher $\frac{{SD\left( {RT} \right)}}{{mean\left( {RT} \right)}}$ ratio values compared to the highest contrast (t(14) = 5.93, *p* = 3.7 × 10^–5^).

Critically, the $\frac{{SD\left( {RT} \right)}}{{mean\left( {RT} \right)}}$ curve was U-shaped as a function of SAT. Indeed, a quadratic fit to the data from each contrast showed a positive quadratic coefficient when averaged across the five contrasts (t(14) = 4.96, *p* = 0.0002). In addition, the quadratic coefficient was significantly positive for all five individual contrasts (contrast 1: t(14) = 5.83, *p* = 4.3 × 10^–5^; contrast 2: t(14) = 3.14, *p* = 0.007; contrast 3: t(14) = 5.9, *p* = 3.9 × 10^–5^; contrast 4: t(14) = 3.48, *p* = 0.004; contrast 5: t(14) = 2.83, *p* = 0.01). The $\frac{{SD\left( {RT} \right)}}{{mean\left( {RT} \right)}}$ curve reached its minimum in the “medium” SAT condition: the $\frac{{SD\left( {RT} \right)}}{{mean\left( {RT} \right)}}$ value for that condition was lower than both the “extremely fast” condition (t(14) = 4.2, *p* = 0.0009) and the “extremely slow” condition (t(14) = 6.56, *p* = 1.3 × 10^–5^). All results above remained virtually unchanged when RT mean and SD were computed using only trials with correct responses. Further, because the standard deviation of RT distributions is very sensitive to outliers, we repeated all analyses using alternative cutoff values of RT and again found very similar results (Supplementary Fig. 4). Finally, the same U-shaped curve was obtained if $\frac{{SD\left( {RT} \right)}}{{mean\left( {RT} \right)}}$ was considered a function not of SAT level but of the mean RT in each SAT condition (Supplementary Fig. 5), and the same relationship can be appreciated when RT standard deviation is plotted directly against RT (Supplementary Fig. 6). These results demonstrate that, contrary to claims regarding a fully linear relationship between mean and SD of RT^19^, varying SAT reveals that the mean and SD of RT distributions have a relationship that systematically deviates from linearity.

#### Skewness of RT distributions

Finally, we explored how SAT modulated the skewness of the RT distributions. A repeated measures ANOVA with factors SAT and contrast revealed a main effect of SAT on skewness values (F(4,56) = 12.9, *p* = 1.6 × 10^–7^) but no main effect of contrast (F(4,56) = 2.39, *p* = 0.06) or interaction between SAT and contrast (F(14,48.7) = 2.37, *p* = 0.79) (Fig. 3D). As before, we tested for the presence of a U-shaped relationship by fitting a quadratic equation to the data for each contrast and found that the quadratic coefficient averaged across the five contrasts was significantly positive (t(14) = 4.89, *p* = 0.0002), indicating a U-shaped curve. Further, the quadratic coefficient was significantly positive for all five individual contrasts (contrast 1: t(14) = 3.63, *p* = 0.003; contrast 2: t(14) = 3.25, *p* = 0.006; contrast 3: t(14) = 2.83, *p* = 0.01; contrast 4: t(14) = 3.06, *p* = 0.008; contrast 5: t(14) = 2.74, *p* = 0.016). The minimum skewness was achieved for the “fast” condition, which had significantly lower skewness compared to both the “extremely fast” (t(14) = 5.74, *p* = 5.1 × 10^–5^) and “extremely slow” (t(14) = 5.99, *p* = 3.3 × 10^–5^) conditions. Virtually the same effects were obtained when skewness was computed using only correct trials. Further, because the skewness of RT distributions is very sensitive to outliers, we repeated all analyses using alternative cutoff values of RT and again found very similar results (Supplementary Fig. 7). Thus, just as the RT difference between error and correct trials and the $\frac{{SD\left( {RT} \right)}}{{mean\left( {RT} \right)}}$ ratio, the RT distribution skewness also exhibited a robustly U-shaped curve as a function of SAT.

### The diffusion model with selective influence assumption cannot fit the data

We observed that varying SAT produces three different U-shaped curves—in the RT difference between error and correct trials, in the ratio of SD and mean of RT, and in the skewness of the RT distribution. These U-shaped curves can be used as strong tests of any theory of RT. Therefore, we examined whether the diffusion model with the selective influence assumption can reproduce these three effects by fitting the model to our data. To do so, we allowed the drift parameter to vary with contrast and the boundary parameter to vary with SAT condition. On the other hand, the non-decision time and three variability parameters were fixed between the different conditions. This parameterization resulted in 14 free parameters. Note that the starting point of the accumulation was always set to halfway between the two boundaries as we did not observe strong response biases.

#### HDDM fits

**Figure 4:**
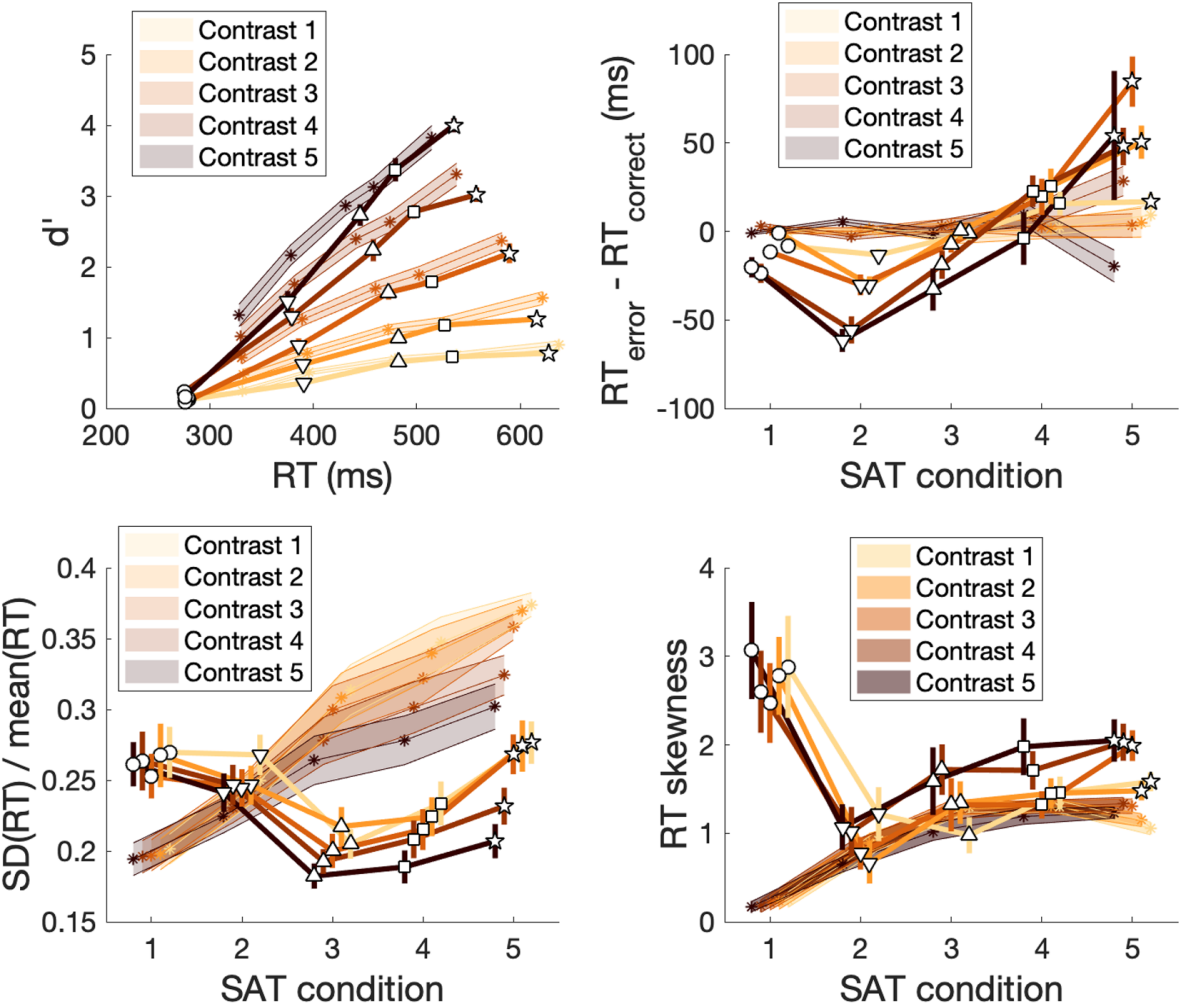
HDDM diffusion model fits. We fit the diffusion model to the data using the software package HDDM by allowing only the boundary parameter to vary with SAT levels and the drift parameter to vary with contrast. The results showed good fits for the d′-RT curves except for the “extremely fast” condition (upper left panel), mostly flat curves for the RT difference between error and correct trials (upper right panel), monotonically increasing $\frac{{SD\left( {RT} \right)}}{{mean\left( {RT} \right)}}$ curves (lower left panel), and inverted-U RT skewness curves (lower right panel). None of the empirically observed U-shaped curves were reproduced even qualitatively. In all subplots, lines represent the empirical data and are identical to what is plotted in Fig. 3. The shaded areas represent models fits and the stars in the shaded areas represent the different SAT conditions. The width of the shaded areas represents S.E.M.

We first fit the diffusion model to the data using what is currently the most popular software package for fitting the diffusion model: the Hierarchical Drift Diffusion Model (HDDM^22^). We did not use the hierarchical feature of the software and instead fit the data for each subject independently keeping all default settings. We found that, perhaps surprisingly, the HDDM fits did not show any of the three U-shaped curves discussed above (Fig. 4).

The HDDM model fits were most successful in reproducing the d′-RT curves from Fig. 3A even though the fits were clearly imperfect, especially for the “extremely fast” condition where the model predicted much higher RT and d′ values that empirically observed. Furthermore, the HDDM fits were not able to capture the pattern of results related to the RT difference between error and correct trials. In fact, the HDDM fits did not show any substantial difference in RT between error and correct trials: except for the “extremely slow” condition for contrast 4 (RT difference = 28 ms) and contrast 5 (RT difference = − 20 ms), all other differences had absolute values smaller than 10 ms indicating HDDM predicts that error and correct trials should have very similar RTs. Similarly, the HDDM fits were not able to reproduce the U-shaped curves for the $\frac{{SD\left( {RT} \right)}}{{mean\left( {RT} \right)}}$ ratio as a function of SAT level and instead exhibited monotonically increasing functions for all contrasts. Finally, the HDDM fits could not reproduce the U-shaped curves for the skewness of the RT distributions as a function of SAT level and exhibited the opposite pattern showing an inverted-U shape as a function of SAT. Overall, the HDDM fits could not replicate any of the three U-shaped functions observed in the data.

#### DMAT fits

The results above show that the HDDM fits deviate substantially from the observed patterns in the data. It is especially startling that the HDDM fits failed to capture the U-shaped curve for the difference between error and correct RT given the central role this quantity has had in the development of the diffusion model^10^. However, a closer look at the way the diffusion model is implemented in HDDM reveals that the failure of the HDDM fits in this particular case may stem from what could seem as a minor technical detail regarding how the parameter $s_{z}$ (which controls the variability of the starting point of the accumulation) varies between conditions. Specifically, when originally introducing the parameter $s_{z}$, Ratcliff and Rouder^15^ fixed its *absolute* value across conditions. On the other hand, HDDM fixes its *relative* size (as a ratio to the height of the boundary $a$) to be constant across conditions^22^. This difference is critical because it affects the relative amount of starting point variability between different SAT conditions. Note that fixing the relative starting point ($\frac{{s_{z} }}{a}$) ensures that the starting point is never beyond the boundaries. On the other hand, fixing the absolute value of $s_{z}$ implies that the boundary $a$ can never go below $2s_{z}$, which may appear to be an undesirable constrained. Presumably due to this reason, two of the most widely used software packages—HDDM^22^ and fast-dm^23^—only allow for the ratio $\frac{{s_{z} }}{a}$ to be fixed across conditions.

**Figure 5:**
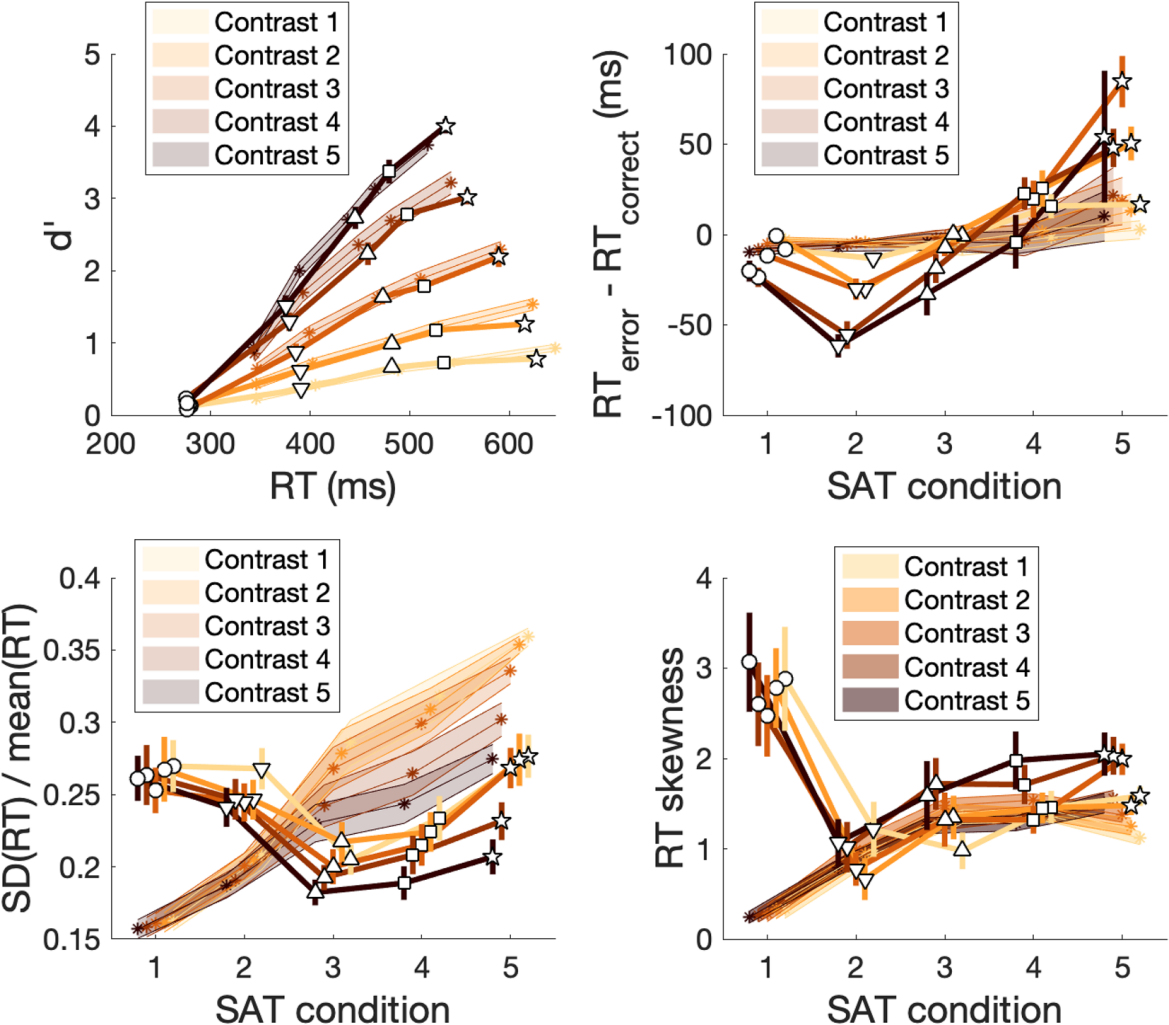
DMAT model fits. We fit the diffusion model to the data using the software package DMAT by allowing only the boundary parameter to vary with SAT levels and the drift parameter to vary with contrast. The fits were very similar to the ones with HDDM (Fig. 4). We observed good fits for the d′-RT curves except for the “extremely fast” condition (upper left panel), monotonically increasing functions for the RT difference between error and correct trials (upper right panel), monotonically increasing $\frac{{SD\left( {RT} \right)}}{{mean\left( {RT} \right)}}$ curves (lower left panel), and inverted-U RT skewness curves (lower right panel). Again, none of the empirically observed U-shaped curves were reproduced even qualitatively. All notation is identical to Fig. 4.

To investigate whether the diffusion model could fit the pattern of U-shaped curves when the absolute value of the parameter $s_{z}$ is kept constant across conditions, we used the Diffusion Model Analysis Toolbox (DMAT^24^) because it keeps the absolute value of $s_{z}$ constant across conditions. We fit our data using DMAT’s default parameters (except for slightly altering the allowable ranges for two parameters; see “Methods”). This fit produced very similar results to the HDDM fit and failed to reproduce any of the three U-shaped curves, including the one for the RT difference between error and correct trials (Fig. 5).

The reason for this poor fit appears to be the fact that, by default, DMAT forces the parameter $s_{z}$ to be smaller than one half of the parameter $a$ (boundary height) in all conditions (this is to prevent the accumulation process from starting beyond either boundary on a particular trial). This constraint creates tension between the “extremely fast” condition where a very low value of the boundary $a$ is needed for a good fit, and the rest of the conditions where a relatively high value of the parameter $s_{z}$ is needed for a good fit. Because $s_{z}$ needs to be lower than $\frac{a}{2}$ in the “extremely fast” condition, DMAT is forced to trade off the values for $a$ and $s_{z}$ thus making $s_{z}$ not large enough for the conditions with a stronger focus on accuracy and making $a$ not small enough for the “extremely fast” condition. Therefore, to remove the need to trade off the values of $s_{z}$ and $a$, we generated custom DMAT fits by changing DMAT’s default settings such that $s_{z}$ was no longer required to be smaller than $\frac{a}{2}$. (We note that, depending on the assumed reasons for the variability of the starting point, allowing $s_{z}$ to be larger than $a$ can be considered nonsensical as that allows the accumulation process to start beyond the boundaries). These custom DMAT model fits still failed to fit well the RT difference between error and correct trials though a qualitatively U-shaped curve emerged (Supplementary Fig. 8). Similar results were obtained if the boundary $a$ and the starting point $s_{z}$ were allowed to vary across SAT conditions but $s_{z}$ was still required to be smaller than $\frac{a}{2}$ (Supplementary Fig. 9).

Finally, we investigated whether the failures of the diffusion model to fit the data stemmed exclusively from the “extremely fast” condition. We used DMAT to fit all data but excluded the data from the “extremely fast” condition. The results (Supplementary Fig. 10) were very similar to the ones obtained in the previous fits (if the “extremely fast” condition is to be ignored). Specifically, the model still could not account for the RT difference between error and correct trials in the “fast condition” or the U-shaped curve for the ratio between the standard deviation and mean of the RT distribution, but could now account well for the RT skewness (which is also true in Figs. 4 and 5 when the “extremely fast” condition is ignored).

In sum, none of the fits were able to reproduce the U-shaped curves observed for the RT difference between error and correct trials, the SD/mean ratio, or RT skewness. Allowing the starting point to either exceed the boundaries or vary with SAT level led to qualitative U-shaped functions that were, however, still unable to capture the data well. Thus, the diffusion model with the selective influence assumption (where SAT should only modulate the decision boundary and difficulty should only modulate the drift rate) failed to account for the observed patterns in the data.

### Possible curves produced by the diffusion model

It can be argued that at least some of the deviations that we observed between the model fits and the data are not inherent to the diffusion model but are simply due to imperfections in the available software for fitting the diffusion model. Further, the fits in Figs. 4 and 5 are averages across many subjects each of which is fitted with a different combination of parameters, which may obscure the true predicted shape of the different curves by a single set of parameters.

To address this potential issue, we examined what possible curves the diffusion model can generate using simulations. In two sets of simulations, we generated diffusion model predictions by fixing the relative or absolute size of $s_{z}$ across conditions. We simulated the diffusion model’s predictions for different sets of parameters in order to explore the range of possible patterns that the model can generate. For all simulations, the boundary $a$ was varied from 0 to 0.2 in steps of 0.01 in order to precisely map out the shapes of the different curves. Further, we fixed the drift rate $v$ to 0.15, the starting point of the accumulation $z$ to halfway between the two boundaries, the non-decision time $T_{er}$ to 0.27, and the non-decision time variability $s_{t}$ to 0.1. All of these values were chosen to roughly match the average performance observed in the data; however, changing any of these values in control simulations produced equivalent results (additional simulations with different parameter values are shown in Supplementary Fig. 11 and reveal the same qualitative results). Critically, we examined how the curves change for different values of drift rate variability $\eta$ (set to 0 or 0.2) and starting point variability $s_{z}$. In the first set of simulations, we fixed $\frac{{s_{z} }}{a}$ to 0 or 0.4; in the second set of simulations we fixed $s_{z}$ to 0 or 0.1. The two values for each of these last three parameters were chosen to be very different from each other in order to obtain a picture of the range of data patterns that the diffusion model can generate.

**Figure 6:**
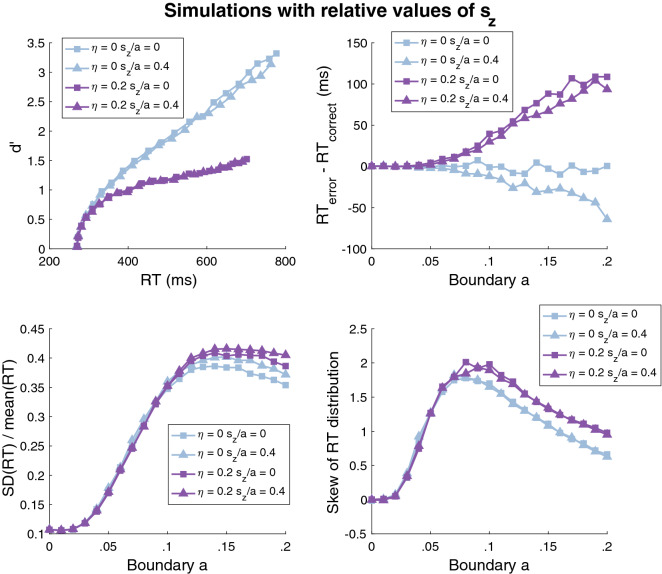
Diffusion model simulations with the relative starting point ($\frac{{s_{z} }}{a}$) fixed across conditions. We simulated the predictions of the diffusion model by keeping $\frac{{s_{z} }}{a}$ constant across conditions. The boundary $a$ varied from 0 to 0.2 in steps of 0.01, the drift rate $v$ was set to 0.15, the starting point of the accumulation $z$ was fixed to halfway between the two boundaries, the non-decision time $T_{er}$ was fixed to 0.27, and the non-decision time variability $s_{t}$ was fixed to 0.1. The simulations produced inverted-U d′-RT curves (upper left panel) even though the empirical curves were linear. For each set of parameters $\left( {\eta ,\frac{{s_{z} }}{a}} \right)$, the points with higher RT correspond to higher values of the boundary $a$. The simulations also produced either monotonically increasing or monotonically decreasing curves for the RT difference between error and correct trials (upper right panel), S-shaped $\frac{{SD\left( {RT} \right)}}{{mean\left( {RT} \right)}}$ curves (lower left panel), and inverted-U shapes for the skewness of the RT distributions (lower right panel). In all of these cases, the qualitative shapes differed from what was observed in the empirical data (Fig. 3).

In the first set of simulations (with $\frac{{s_{z} }}{a}$ constant across conditions), the diffusion model produced d’-RT curves that consistently had inverted-U shapes (as measured by the sign of the quadratic coefficient in a quadratic fit; Fig. 6, upper left panel), which were mostly driven by low values of the boundary $a$. More critically, the diffusion model could not generate U-shaped curves for the RT difference between error and correct trials (Fig. 6, upper right panel). In fact, the diffusion model only produced monotonically increasing or monotonically decreasing curves of RT difference as a function of SAT. In other words, for any combination of parameters, changing the boundary could only affect the size of the RT difference between error and correct trials but not change its sign. Thus, when $\frac{{s_{z} }}{a}$ is kept constant across conditions, the diffusion model cannot fit any data where two conditions that only vary in SAT produce RT differences of different signs. Further, the diffusion model could not produce the U-shaped curves for $\frac{{SD\left( {RT} \right)}}{{mean\left( {RT} \right)}}$ and RT skewness as a function of SAT (Fig. 6, lower panels). Instead, the model produced S-shaped curves for $\frac{{SD\left( {RT} \right)}}{{mean\left( {RT} \right)}}$ and inverted-U shapes for skewness. Therefore, the failure of HDDM to fit the three U-shaped curves when $\frac{{s_{z} }}{a}$ is kept constant across conditions appear to be fundamental to the structure of the diffusion model and is not simply the product of the specific implementation of the model in the HDDM package.

**Figure 7:**
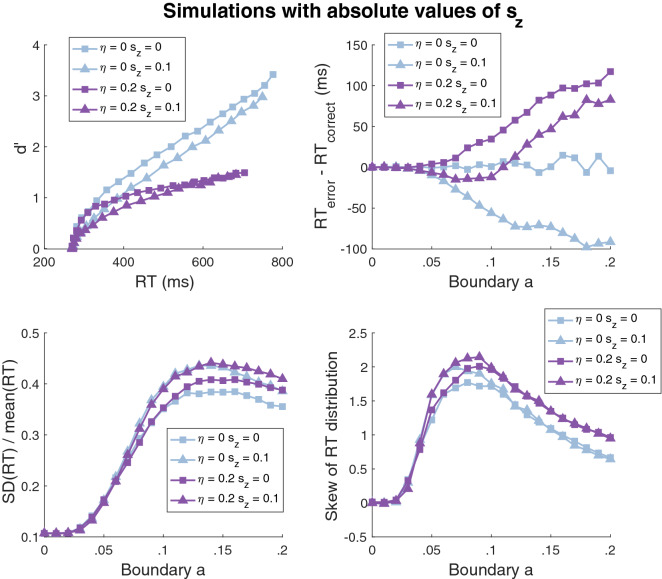
Diffusion model simulations with absolute starting point ($s_{z}$) fixed across conditions. We fit the diffusion model to the data with $s_{z}$ constant across conditions. All other details were the same as in the simulations from Fig. 6. The simulations produced virtually equivalent results as when fixing $\frac{{s_{z} }}{a}$ across conditions, except that U-shaped curves emerged when the parameters $\eta$ and $s_{z}$ both had relatively large positive values.

We then considered the second set of simulations (with $s_{z}$ constant across conditions). We found virtually the same results for the d’-RT, $\frac{{SD\left( {RT} \right)}}{{mean\left( {RT} \right)}}$, and RT skewness curves. Specifically, just as with the first set of simulations, we obtained inverted-U-shaped curves for all d’-RT functions (Fig. 7, upper left panel), S-shaped $\frac{{SD\left( {RT} \right)}}{{mean\left( {RT} \right)}}$ curves (Fig. 7, lower left panel), and strongly inverted-U-shaped curves for RT skewness (Fig. 7, lower right panel). However, the two sets of simulations differed in the predicted shapes of the curves for the RT difference between error and correct trials. Just as in the first set of simulations, setting either the drift rate variability ($\eta$) or starting point variability ($s_{z}$) to zero produced monotonically increasing or decreasing functions (Fig. 7, upper right panel). Critically, however, if both parameters were positive (and they were both within reasonable ranges so that one of them did not dominate the other), then a U-shaped function emerged. This finding shows that for U-shaped curves to emerge for the RT difference between error and correct trials, it is necessary that $s_{z}$ rather than $\frac{{s_{z} }}{a}$ is kept constant across conditions, which, however, means that the starting point will exceed the boundaries when the boundary parameter takes very small values. The simulations also show that, as currently constructed, the diffusion model appears to never result in U-shaped curves for $\frac{{SD\left( {RT} \right)}}{{mean\left( {RT} \right)}}$ or RT skewness.

### Fitting each condition with the diffusion model independently

Finally, we used our data to test the “selective influence” assumption that different SAT settings should only affect the boundary $a$, whereas different difficulty levels should only affect the drift rate $v$^21^. We fit each condition of the experiment (that is, each combination of contrast and SAT level) independently of all others and examined how the different model parameters changed. Each condition was thus fit with six free parameters: drift rate, boundary, non-decision time, drift rate variability, non-decision time variability, and starting point variability (as before, the starting point was set to always be halfway between the two boundaries). These very flexible fits allowed the diffusion model to do a better job at capturing the qualitative effects in the data but the fits remained imperfect even when all parameters could vary across all conditions (Supplementary Fig. 12).

We next tested the selective influence assumption of the model. Because the “extremely fast” SAT condition resulted in chance (or near chance) performance, we only analyzed the last four SAT conditions (“fast,” “medium,” “slow,” and “extremely slow”), as well as all the five contrast levels. For each of the six free diffusion model parameters, we performed a repeated measures ANOVA with factors contrast and SAT (we performed seven sets of tests because we examined $s_{z}$ and $\frac{{s_{z} }}{a}$ separately). We considered the main effects of contrast and SAT, as well as their interaction. To obtain the fits, we used both HDDM and DMAT. Below we report the HDDM results; the DMAT results were similar (Supplementary Fig. 13).

**Figure 8:**
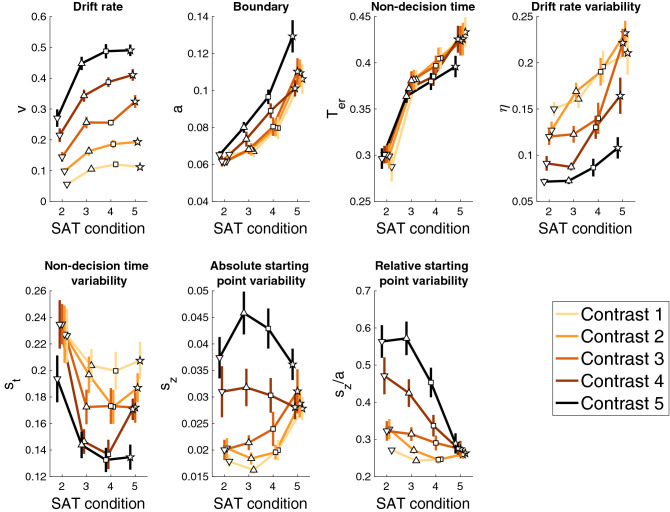
Dependence of each diffusion model parameter on contrast and SAT. We fit each combination of contrast and SAT level (except for the “extremely fast” SAT condition) with the diffusion model independently from all other conditions. We then examined how each parameter of the diffusion model depends on contrast and SAT. We found that contrary to the diffusion model predictions, all parameters changed with both contrast and SAT levels. All notation is identical to Fig. 3.

Contrary to the “selective influence” assumption, we observed a significant effect of both contrast and SAT on every single one of the six parameters (Fig. 8). Specifically, we found significant effects of contrast on the drift rate (F(4,56) = 303.25, *p* = 3.1 × 10^–37^), boundary (F(4,56) = 7.56, *p* = 6.1 × 10^–5^), non-decision time (F(4,56) = 12.75, *p* = 1.9 × 10^–7^), drift rate variability (F(4,56) = 63.7, *p* = 3.5 × 10^–20^), non-decision time variability (F(4,56) = 18.25, *p* = 1.2 × 10^–9^), absolute starting point variability (F(4,56) = 36.28, *p* = 6 × 10^–15^), and relative starting point variability (F(4,56) = 41.93, *p* = 3.2 × 10^–16^). Similarly, we found significant effects of SAT on the drift rate (F(3,42) = 54.71, *p* = 1.5 × 10^–14^), boundary (F(3,42) = 63.78, *p* = 1.1 × 10^–15^), non-decision time (F(3,42) = 40.76, *p* = 1.7 × 10^–12^), drift rate variability (F(3,42) = 19.16, *p* = 5.5 × 10^–8^), non-decision time variability (F(3,42) = 7.46, *p* = 0.0004), and relative starting point variability (F(3,42) = 15.06, *p* = 8.4 × 10^–7^) though there was no significant effect on absolute starting point variability (F(3,42) = 2.13, *p* = 0.11). Finally, and again contrary to the diffusion model predictions, there was a significant interaction between contrast and SAT for all six parameters (drift rate: F(14,52.9) = 4.27, *p* = 2 × 10^–13^, boundary: F(14,54.8) = 4.27, *p* = 0.0006, non-decision time: F(14,42.6) = 4.65, *p* = 2.3 × 10^–5^, drift rate variability: F(14,42.9) = 2.44, *p* = 0.001, non-decision time variability: F(14,45) = 1.72, *p* = 0.01, absolute starting point variability: F(14,42) = 2.87, *p* = 0.0002, and relative starting point variability: F(14,37.9) = 1.87, *p* = 2.6 × 10^–8^).

Finally, we compared the effects sizes of our contrast and SAT manipulations on different parameters. We calculated the change from the “fast” to the “extremely slow” SAT conditions, as well as for the change from contrast 1 to contrast 5. Note that an absolute value of Cohen’s d larger than 0.8 is considered a “large” effect size. Surprisingly, the SAT manipulation had a slightly larger effect on the drift rate (Cohen’s d = 2.89) than on the boundary (Cohen’s d = 2.45). It also had large effects on many other parameters (non-decision time: Cohen’s d = 1.85; drift rate variability: Cohen’s d = 1.49; non-decision time variability: Cohen’s d = − 0.7; absolute starting point variability: Cohen’s d = 0.48; relative starting point variability: Cohen’s d = − 1.24). On the other hand, the contrast manipulation had the largest effect size on the drift rate parameter (Cohen’s d = 5.29) but the effects on the rest of the parameters were also quite large (boundary: Cohen’s d = 0.74; non-decision time: Cohen’s d = − 1.26; drift rate variability: Cohen’s d = − 3.19; non-decision time variability: Cohen’s d = − 2.24; absolute starting point variability: Cohen’s d = 2.27; relative starting point variability: Cohen’s d = 2.37). Therefore, both the SAT and contrast manipulations had large effects on all parameters. In the case of SAT, the largest effect was in fact on drift rate rather than the boundary. These results strongly question the “selective influence” assumption of the diffusion model.

## Discussion

How people trade off speed for accuracy has been the subject of intense research for over 50 years^2,25,26^. However, despite substantial progress, it remains unclear what the signatures of SAT are beyond the mere change in mean RT and accuracy. Here we describe three fundamental effects of SAT. Specifically, we find that SAT produces U-shaped curves for the difference between error and correct trials, the ratio of SD to mean of the RT distribution, and the skewness of the RT distribution. These U-shaped curves could be observed in individual subjects and were present across different levels of contrast. Our results thus establish a challenging set of benchmark tests for models of RT. Importantly, the diffusion model with a selective influence assumption could not reproduce any of these effects. Moreover, allowing all parameters to change across conditions demonstrated the presence of large and systematic changes in all diffusion model parameters with both SAT and difficulty.

### RT difference between error and correct trials

The difference in RT between error and correct trials has been a driving force in the development of the diffusion model^9,10,15^. In fact, two of the seven diffusion model parameters have been specifically introduced in order for the model to be able to reproduce observed differences between error and correct RTs^10^. Nevertheless, to our knowledge, the observed U-shaped curve for the RT difference between error and correct trials has not been explicitly described before. Indeed, the great majority of previous research, including our own work, only compared two levels of SAT^27–29^ but such studies are not in a position to describe the whole curve. At the same time, many other studies did generate a large number of SAT levels, and, in some cases, there were even more such conditions that the current study^25^. However, these studies did not examine the difference in RT between error and correct trials (and, in many cases, they did not include a sufficient number of subjects to perform group-level statistics).

This is not to say that the U-shaped curve for the RT difference between error and correct trials is completely novel; in fact, it could have already been deduced by the prior literature. Indeed, it is well known that in the absence of speed stress, error RTs are longer than correct RTs, whereas in the presence of strong speed stress, error RTs become shorter than correct RTs^14^. On the other hand, not many studies have examined what happens under extreme speed stress where performance falls to chance but the results here are easy to anticipate: if performance is at chance, then there is no perceptual signal for the observer to work with, and therefore correct and error trials must occur randomly. It logically follows that there is no difference in how correct and error trials are generated and thus such extreme speed stress would produce the same mean RT for both trial types (and this is indeed what we observed). Putting these three effects together, we see that the difference in RT between error and correct trials must start at zero for extreme speed stress, then become negative for strong but not extreme speed stress, and finally become positive when speed stress is completely eliminated. In other words, the U-shaped curve observed in the present study is already implied by the previous literature.

What gives rise to the U-shaped curve for the difference between error and correct RTs? As described above, extreme speed stress (that results in chance-level performance) necessarily leads to no RT difference between error and correct RTs. We speculate that as the speed stress is relaxed slightly, there is trial-to-trial variability in the internal response deadline set by subjects. In this regime of strong speed stress, trials that happen to have a stricter internal deadline are more likely to be fast but also likely to be incorrect as less information was processed. This difference leads to error trials being faster than correct trials. As speed stress is completely lifted, the RT difference between error and correct trials is driven by a different phenomenon—stochastic variability in trial difficulty. Such variability in difficulty makes some trials more difficult and thus leads to higher RTs and lower accuracy. In this regime of no speed stress, error trials thus become slower than correct trials.

Note that the mechanisms that govern RT differences between error and correct trials in the diffusion model agree with the above explanation in the case of no speed stress but not for the above explanation for the case of strong speed stress. Indeed, in the case of no speed stress, the diffusion model generates a difference between error and correct RTs based on the parameter $\eta$ (drift rate variability), which essentially introduces stochastic differences in trial-by-trial difficulty. However, the intuition above about stochasticity of the internal deadline for response in the presence of strong speed stress would correspond to variability in the location of the boundary parameter $a$, whereas the diffusion model eschews variability in this parameter and instead introduces it in the starting point of the accumulation. It is an open question whether a version of the diffusion model with variability in the boundary rather than the starting point would be better able to fit the effects of strong speed stress on RTs.

### RT distribution SD/mean ratio

As mean RT is increases, the standard deviation of the RT distribution also increases. It is therefore natural to examine the relationship between the rates of increase for these two quantities. In an influential paper, Wagenmakers and Brown^19^ examined the relationship between SD and mean of RT distributions constructed in several different ways and found very high correlations (often r > 0.9). The authors consequently argued that such high correlations appear to reveal what amounts to a psychophysical law of linear SD-mean relationship. The conclusion is perhaps surprising given that at least half of the individual datasets examined appeared to have the same systematic deviation from linearity where the SD for intermediate values of mean RT was higher than the linear fit would suggest. In addition, Wagenmakers and Brown never examined how the SD-mean relationship is specifically influenced by SAT. Instead, for all analyses, they correlated the RT mean and SD for all conditions taken together. This type of analysis, however, can mask non-linearities produced by a given manipulation. Indeed, when we performed the same type of analysis on the data from our experiment (by creating 25 conditions obtained by combining the five SAT levels with the five contrast levels), we observed very high correlations between RT mean and SD; the correlation coefficient r exceeded 0.85 for half of the subjects and exceeded 0.7 for 17 of the 20 subjects. Therefore, very high correlations in analyses that combine all conditions of an experiment can be obtained even in the presence of manipulations that produce non-linear effects (such as the SAT manipulation in the current experiment).

Our results demonstrate that SAT manipulations lead to nonlinear effects in the relationship between mean and SD of the RT distributions. This nonlinearity is best described by plotting the shape of the SD/mean ratio as a function of either SAT level (Fig. 3C) or mean RT (Supplementary Fig. 5). These plots show a robustly U-shaped relationship that has not been described before.

What gives rise to the U-shaped curve of the SD/mean ratio as a function of SAT level? At the moment, we can only speculate. One way to describe the observed phenomenon is that the variability of the RT distribution is smallest, relative to its mean, for intermediate levels of speed stress. This formulation of the phenomenon suggests that the cause may be driven by an increased consistency in response strategy and execution with intermediate but not extreme speed stress. However, at the moment, this explanation is little more than restating the observed effect; the actual mechanisms of the proposed increase in response consistency remain to be elucidated.

### Skewness of RT distributions

Fitting the exact shape of the RT distributions has long been the stated goal of the diffusion model, as well as other models from the sequential sampling framework^8^. In fact, the diffusion model is thought to fit precisely the shape of individual RT distributions^10^. Moreover, it has been claimed that the diffusion model can fit well true RT distributions but not fake ones^20^.

However, this line of research has rarely quantified higher moments such as the skewness or kurtosis (but see, for example^30^), especially as a function of SAT level. Instead, what is typically considered are the fits to specific characteristics of the distribution like different percentiles or the leading edge of the distribution^31^. Our results demonstrate that the third moment of the RT distribution—its skewness—produces a characteristic U-shaped curve as a function of SAT. Higher moments are very sensitive to outliers but our analyses showed that the shape of the curve is virtually unchanged when different RT cutoffs are used (Supplementary Fig. 7).

What is the reason for the U-shaped curve of RT skewness as a function of SAT level? It is again difficult to speculate at this point. One possibility is that the U-shaped curve of RT skewness is caused by similar processes as the ones that give rise to the U-shaped curve for the SD/mean ratio of the RT distribution. However, the minimum for the SD/mean curve was consistently reached for the “fast” SAT condition, whereas the minimum for the skewness curve was consistently reached for the “medium” SAT condition. Therefore, it is unlikely that the exact same process gives rise to the U-shaped curves for both the SD/mean ratio and skewness. It is thus likely that multiple mechanisms related to the effects of SAT on the RT distribution remain to be discovered.

### The inability of the diffusion model to account for the shape of the observed curves

Both our fitting and simulation results demonstrated that the current diffusion model with the selective influence assumption cannot explain the observed U-shaped curves for the RT difference between error and correct trials, the SD/mean ratio, and skewness of the RT distribution. These deviations between data and model are substantial since they are qualitative and not just quantitative. In fact, it can be argued that, collectively, the presence of so many severe deviations between the data and the diffusion model suggests that the model does not capture the dynamics of evidence evaluation in our task. It remains an open question whether other models from the sequential sampling framework can explain the observed patterns of results though additional analyses with the linear ballistic accumulator (LBA) model produced even worse fits and also could not account for any of the three U-shaped curves (Supplementary Figs. 14, 15).

One important question is to what extent our results are specific to the task that we used. Our task featured a perceptual judgment following a very brief stimulus presentation (of only 33 ms). It could be that the diffusion model does not apply well to such short presentations and only works well when the stimuli are presented for an extended period. For example, short stimulus presentations may violate the diffusion model’s assumptions that evidence arrives for the duration of decision making and that different samples are not autocorrelated. Nevertheless, current theories do not make a distinction between the diffusion model’s ability to explain the processes underlying evidence evaluation in short vs. long stimulus presentations^10,31^. In fact, some of the most influential papers in the field have used very short stimulus presentations that are as brief as 12 ms^32^. Further, prominent researchers have explicitly argued that very short stimulus presentations induce a short-term representation that provides a constant drift rate to drive the decision process, and that the diffusion model is an appropriate model for such cases^31^. Therefore, our experimental design fits with many other studies on the diffusion model. Nevertheless, it remains possible that the diffusion model should only be applied to specific paradigms and that short stimulus presentations are not amongst them. However, we note that such a conclusion would necessitate a re-examination of a substantial portion of the existing literature.

A natural question is whether an extension of the diffusion model can accommodate some of the observed effects. One possibility is that adding variability in the response boundary could allow the model to explain some of the observed U-shaped curves. (The boundary is currently the only “major” parameter of the diffusion model that is not hypothesized to vary between trials). To check for this possibility, we performed several simulations of the model with added variability to the boundary parameter but failed to obtain U-shaped curves for SD/mean or the skewness of the RT distributions. More systematic efforts are needed in this direction but at the moment it appears that the addition of one more variability parameter would be insufficient to explain the results obtained in the present paper.

One specific way in which the diffusion model fails to account for the data is in the skewness of the RT distribution in the “extremely fast” condition. In that condition, performance is virtually at chance, which is naturally accommodated by the diffusion model by setting the boundary extremely low. In this regime, boundary-crossing occurs almost instantaneously and thus the shape of the RT distribution is determined almost exclusively by the variability of the non-decision time. Since non-decision time is modeled as having a uniform distribution^16^, one naturally obtains skewness values of zero (or close to zero) when simulating such a condition with the diffusion model (see Figs. 6, 7). However, our data show that this condition produced substantial RT skewness (Fig. 3D). To accommodate this effect, the diffusion model should be modified to include non-decision time with variability which is itself a right-skewed function. In fact, previous work has suggested that non-decision times are likely to have a substantial amount of right skew^33^. Further, this research demonstrated that estimating the true shape of the distribution of non-decision times changes substantially the diffusion model’s fits and, in some cases, alters the conclusions that could be drawn from these fits. Nevertheless, it remains to be tested whether substituting the uniform distribution of non-decision time with a right-skewed one will allow the model to reproduce any of the curves in our study. In fact, informal simulations with non-decision time drawn from a Gamma distribution suggested that changing the variability of the non-decision time can change the shape of the SD/mean and skewness curves but never produces the U-shaped functions observed in the empirical data. Thus, it appears at the moment that no straightforward extension of the diffusion model can accommodate our results regarding the shape of the d′-RT, the ratio of the SD to mean of the RT distribution, or the skewness of the RT distribution. Nevertheless, we acknowledge that there are many possible diffusion model extensions including models with extra parameters for fast guesses^34^, collapsing boundaries^35–37^, within-trial variability that changes across conditions^38^, separate time-based accumulators^39^, etc. It is possible that a model with one or more of these extensions would be able to fit one or more of the qualitative patterns described here but we have not investigated such extensions here.

### The nature of the “extremely fast” SAT condition

Two of the U-shaped functions (the difference between error and correct RT, Fig. 3B, and the skewness of the RT distribution, Fig. 3D) owe their U shape exclusively to the “extremely fast” condition. It is reasonable therefore to wonder whether this condition may be an outlier that should be excluded. Indeed, the condition could be seen as a pure detection task and thus different from the remaining SAT conditions. Our results contradict such a conclusion because performance in the “extremely fast” condition was above chance and the condition appears to lie on the same continuum as the rest of the conditions (Supplementary Fig. 3). Moreover, excluding this condition would not change the conclusions for the $\frac{{SD\left( {RT} \right)}}{{mean\left( {RT} \right)}}$ ratio (Fig. 3C) and will lead to a conclusion that the RT difference between error and correct should be monotonically increasing, which contradicts both logical considerations (see “Discussion” above on why U-shaped functions for the RT difference between error and correct trials occur) and the diffusion model itself (see Figs. 6, 7). Due to these reasons, we believe that the “extremely fast” condition provides meaningful results that can reasonably be considered to be within the purview of the diffusion model. Nevertheless, we have run all diffusion model analyses after excluding the data from the “extremely fast” condition and found that no conclusion changes except for the inability of the diffusion model to explain the RT skewness results (Supplementary Fig. 10). That said, it remains possible that extreme speed stress results in a pattern of subject behavior that is qualitatively different from conditions with less severe speed stress, and that extreme speed pressures should be considered outside of the scope of the diffusion model.

### Selective influence in the diffusion model

The diffusion model is thought to capture meaningful latent variables that cannot be recovered without model fitting^10^. The two most prominent such latent variables are drift rate, which is conceptualized as corresponding to stimulus sensitivity, and boundary separation, which is conceptualized as corresponding to the SAT setting. Therefore, it has been hypothesized that SAT manipulations should only affect the boundary parameter, whereas difficulty manipulations should only affect the drift rate parameter. The existence of such one-to-one mapping between task manipulations and diffusion model parameters has been referred to as “selective influence”^21^.

The selective influence hypothesis has been examined in a number of previous studies with mixed results^40,41^. Typically, studies examined the effects of a particular manipulation on a subset of parameters only, and typically the manipulation consisted of two levels (e.g., fast vs. accurate SAT setting or easy vs. difficult condition) rather than the more detailed five levels used in the present study. Nevertheless, even with these caveats, previous studies found a number of deviations from the selective influence assumption such as speed emphasis leading to decreased non-decision times^40–43^, decreased drift rate variability^44^, and decreased drift rates^42,44–48^. Moreover, easier stimuli were found to decrease the non-decision time, increase the starting point of the accumulation, and increase the decision boundary^41^. The majority of these papers took the approach that was also adopted in the current paper: allow many parameters to vary across different conditions and then use statistical tests to compare the values of the best fitting parameters across conditions. Within the context of this approach, our results show what are perhaps the most severe deviations from the selective influence assumptions observed to date. We observed that both SAT and difficulty manipulations affected every single one of the six model parameters in the diffusion model (the seventh parameter—the starting point of the accumulation—was fixed across conditions and therefore the selective influence assumption was not tested for it). Further, the effects of these two manipulations on the “wrong” parameters were often quite large, and, in the case of the SAT manipulation, the effect on drift rate was slightly larger than on the boundary. On the other hand, it should be noted that our approach does not test for the necessity of different parameter changes, which requires comparing models that allow different combinations of parameters to change across conditions.

Our results raise the question about the reasons for the observed violations of the selective influence hypothesis. Some of the effects observed in our study have been described before (e.g., speed emphasis decreasing non-decision times^40–43^, drift rate variability^44^, and drift rates^42,44–48^) and in many cases psychological mechanisms have been proposed to explain such findings. It is indeed possible that some of these effects reflect true psychological effects (e.g., people may be able to decrease their sensory or motor processing times under speed pressure). However, in the absence of an independent way of measuring different psychological constructs, such proposals remain speculative. We uncover substantial modulations of all diffusion model parameters with both contrast and SAT manipulations, and therefore it would be especially challenging to interpret these modulations as the result of meaningful psychological effects (though some of the modulations may very well be meaningful). Instead, our findings raise doubts about whether the psychological constructs that the diffusion model seeks to uncover map well onto individual diffusion model parameters.

### U-shaped curves as new benchmark tests for models of RT

Evaluating individual models of decision making is difficult^49^. Many techniques are available for the comparison of two or more models^50^ but it is less clear how individual models can be evaluated in isolation. Recently, Oberauer et al.^51^ collected a number of benchmarks for models of short-term and working memory to help guide model development in that domain. Unfortunately, it is unclear what findings related to RT can be used as similar benchmarks in the field of decision making. Several papers have made brief lists of possible benchmarks, often in the context of what the diffusion model can already explain^8^. However, benchmark results differ in their difficulty—some benchmarks are rather easy to meet, while others are very challenging.

It seems uncontroversial to say that U-shaped (or inverted-U-shaped) functions are generally more difficult to reproduce than monotonic ones. Therefore, we suggest that the three U-shaped curves found here can serve as especially challenging benchmarks for current and future models of decision-making RTs. Nevertheless, we caution that these benchmarks should ideally be replicated using different stimuli and tasks as some of them may be specific to perceptual decision making and not to, for example, memory. (However, for reasons explained above, we believe that the U-shaped curves for the RT difference between error and correct trials can already be adopted as a robust benchmark.)

## Conclusion

Using detailed manipulations of SAT and task difficulty in a sample of 20 subjects with 5000 trials/subject, we uncovered three fundamental effects wherein SAT manipulations resulted in U-shaped curves for (i) the RT difference between error and correct trials, (ii) the SD/mean ratio of the RT distributions, and (iii) the skewness of the RT distributions. These findings can serve as challenging benchmarks for testing models of RT. Critically, the diffusion model with a selective influence assumption could not account for any these benchmarks. Overall, our findings have several important implications about the diffusion model. First, even though the model was specifically designed to fit the RT difference between error and correct trials, it could not fit the RT difference between error and correct trials in the current study unless unorthodox assumptions were made (and, even then, the fit is qualitatively but not quantitatively correct). Second, the claimed psychophysical law proposed by Wagenmakers and Brown^19^ of linearity between SD and mean of RT, which has been argued to provide strong support for the diffusion model, breaks down with SAT manipulations. This fact eliminates one of the most impressive successes of the diffusion model. Third, our results show that the selective influence assumption of the diffusion model does not simply fail for some model parameters under some manipulations, as previous research has suggested. Instead, when sufficient amount of data is collected, the two most fundamental manipulations (SAT and difficulty) each impact every single one of the diffusion model parameters. This finding casts doubt on the fidelity with which the diffusion model is able to reveal meaningful latent variables. Overall, our study shows that the limitations of the diffusion model extend farther than previously appreciated and that the model simply does not provide an appropriate explanation of RT under SAT manipulations.

## Methods

### Subjects

Thirty healthy subjects with normal or corrected to normal vision were recruited and completed one session of testing. Ten subjects were not invited for the rest of the experiment and excluded from further analyses since either they had difficulties in generating five distinct levels of speed stress. None of the analyses reported in the paper were used as exclusion criteria and were never performed on data from the first session alone. The remaining twenty subjects completed all five sessions of the experiment (13 females, age range: 18–28). All subjects signed informed consent and were compensated for their participation. The protocol was approved by the Georgia Institute of Technology Institutional Review Board. All methods were carried out in accordance with relevant guidelines and regulations.

### Task

Subjects performed an orientation discrimination task where they indicated whether a Gabor patch (radius = 4°) embedded in random pixel noise was tilted counterclockwise (“left”) or clockwise (“right”) from vertical. The orientation of the Gabor patch was ± 45° away from the vertical and was randomly determined on each trial. Immediately, after providing their response, subjects were given detailed feedback that indicated (i) whether their response was correct or wrong, (ii) the exact response time on the trial, (iii) the points gained or lost on the trial, and (iv) the total accumulated points until that point of the experiment (Fig. 2).

Each trial started with subjects fixating on a small white dot at the center of the screen for 1000 ms and was followed by a presentation of the Gabor patch for 33 ms. There was no deadline on the response but large RTs were penalized in all conditions except for the “extremely slow” condition. Feedback remained on the screen until the subject pressed a button on the keyboard to advance to the next trial.

The experiment included five different speed-accuracy tradeoff conditions—“extremely fast,” “fast,” “medium,” “slow,” and “extremely slow.” Each condition featured a different penalty on response time (RT) starting from no penalty at all in the “extremely slow” condition and increasing the size of the penalty for the other four conditions. Based on the accuracy and RT on each trial, subjects gained points based on the following formula:

$$ Points \,gained = Acc - penalty_{cond} \times RT + adjustment_{cond} , $$

where $Acc$ is the accuracy on the current trial (1 for correct responses, 0 for wrong responses), $penalty_{cond}$ and $adjustment_{cond}$ are the penalty magnitude on RT and adjustment for the particular condition. The parameter $penalty_{cond}$ was set to 4, 2, 1, 0.5, and 0, respectively for the five conditions with decreasing speed stress. To ensure that the five conditions resulted in relatively similar earnings for the subjects, we added the parameter $adjustment_{cond}$ which added or subtracted a fixed number of points on each trial such that conditions with greater speed stress received the largest positive adjustments. Specifically, the value of $adjustment_{cond}$ was set to + 1, + 0.3, 0, − 0.2, and − 0.5, respectively for the five conditions with decreasing speed stress. Finally, we wanted to prevent a strategy where a subject simple held the keyboard button pressed even before the stimulus appeared. Therefore, a response time of less than 34 ms (roughly corresponding to the duration of the stimulus presentation itself) was penalized by subtracting 3 points; such trials were accompanied with a feedback screen that read “Looks like you held the button before the stimulus appeared” coupled with information about the 3 points lost and the total number of points so far. At the end of the experiment, every point was rewarded with 1 cent of bonus payment.

### Procedure

Subjects came for five different sessions each consisting of 1000 experimental trials. Before the start of the first session, subjects were given detailed instructions about the different conditions and their associated formulas for gaining points. It was specifically emphasized that the best strategy in the “extremely fast” condition was to respond as quickly as possible irrespective of accuracy. This instruction was given explicitly because the majority of subjects appeared unwilling to guess randomly for all trials of a given condition without being explicitly told that such a strategy was allowed. Before the start of sessions 2–5, subjects were briefly reminded of these instructions. Each session started with a short training which included one 25-trial block of each of the five conditions.

In each session, subjects completed 4 runs, each consisting of 5 blocks of 50 trials each. Each block consisted of a single condition, and each run included blocks from all five different conditions in a randomized order. At the beginning of each block, subjects were given the name of the condition for that block (“extremely fast,” “fast,” “medium,” “slow,” or “extremely slow”). In each block, we pseudo-randomly interleaved trials with five different Gabor patch contrasts such that each contrast was presented exactly 10 times. The contrasts were the same for each subject and were chosen based on pilot testing to produce a range of performance levels. The exact contrasts used were 4.98%, 6.39%, 8.21%, 10.54%, and 13.53% (the contrasts were chosen to be equally spaced in log space: they are equal to $e^{ - 3}$, $e^{ - 2.75}$, $e^{ - 2.5}$, $e^{ - 2.25}$, and $e^{ - 2}$, respectively).

### Apparatus

The experiment was designed in MATLAB environment using Psychtoolbox 3^52^. The stimuli were presented on a 21.5-inch iMac monitor (1920 × 1080 pixel resolution, 60 Hz refresh rate) in a dark room. Subjects were seated 60 cm away from the screen and provided their responses using a computer keyboard.

### Behavioral analyses

We removed all trials with RTs shorter than 150 ms or longer than 1500 ms. This step resulted in removing an average of 2.3% of total trials (range 0.3–4.7% for each subject). Similar results were obtained if RT outliers were instead determined separately for each condition using Tukey’s interquartile criterion. We then computed, for each combination of SAT condition and Gabor patch contrast, the average difference between error and correct RTs ($RT_{error} - RT_{correct}$), the median RT for each condition, the ratio of the standard deviation and the mean RT ($\frac{{SD\left( {RT} \right)}}{{mean\left( {RT} \right)}}$), and the skewness of the RT distribution. The skewness was computed as Pearson’s moment coefficient of skewness, which is equal to $\frac{{E\left[ {\left( {X - \mu } \right)^{3} } \right]}}{{\sigma^{3} }}$ where $\mu$ and $\sigma$ are the mean and standard deviation of the distribution, respectively. In addition, we computed the signal detection theory parameter *d*′ that quantifies stimulus sensitivity in signal-to-noise units^53^. Specifically, *d*′ was computed by treating correctly judged clockwise orientations as hits and applying the formula:

$$ d^{\prime} = \Phi^{ - 1} \left( {hit\, rate} \right) - \Phi^{ - 1} \left( {false \,alarm \,rate} \right), $$

where $\Phi^{ - 1}$ is the inverse of the cumulative standard normal distribution that transforms HR and FAR into z-scores.

We quantified the quadratic trends of different curves produced by the five different SAT conditions by fitting a quadratic model $y = ax^{2} + bx + c$. The model was fit separately to each contrast of each subject. Group-level t-tests were then performed on the quadratic coefficients obtained for each subject. Statistical tests were based on two-sided one-sample t-tests, paired t-tests, and repeated measures ANOVAs.

### Diffusion model analyses

We fit the diffusion model^10^ to the data using both the hierarchical drift diffusion model (HDDM) python package^22^ and the diffusion model analysis toolbox (DMAT) in MATLAB^24^. For both packages, all fits were done on the data for each subject (the hierarchical option of HDDM was not used). We performed two different sets of model fitting. In the first model fitting, we let the drift rate parameter vary for different contrasts and the boundary parameter vary for different SAT condition (and fixing all other parameters across contrasts and conditions). In the second model fitting, we let all diffusion model parameters other than the starting point (which was always fixed halfway between the two boundaries) vary with both Gabor patch contrast and SAT condition.

The first set of model fittings—which we refer to as the “constrained” fits—were designed to test whether the diffusion model can account for the patterns of the data using the selective influence assumption that stimulus difficulty should only affect the drift rate and that the SAT setting should only affect the boundary^10^. For these fits, we fit a different drift rate parameter for each of the five Gabor patch contrasts, and a different boundary parameter for each of the five SAT conditions. We fixed all other parameters to be the same across contrasts and SAT conditions. The non-decision time and three variability parameters were estimated as part of the model fitting, whereas the starting point was not treated as a free parameter but was fixed to half the value of the boundary. This parameterization resulted in 14 free parameters (5 drift rates, 5 boundary parameters, and 4 additional parameters that were the same across contrasts and conditions). To evaluate whether these “constrained” fits could account for the patterns observed in the data, once parameter fits were obtained, we generated simulated data based on the recovered parameters. For each subject, we simulated 1000 data points for each combination of contrast and SAT condition (for a total of 25,000 simulated data points per subject). We then analyzed these simulated data in the same way as the analyses of the original subject data.

It should be noted here that HDDM and DMAT treat the parameters for the starting point of the accumulation ($z$) and the starting point variability ($s_{z}$) differently from each other. HDDM defines both of these parameters as a proportion of the value of the boundary parameter $a$. Therefore, when using HDDM, it is only possible to fix the ratios $\frac{z}{a}$ and $\frac{{s_{z} }}{a}$ across conditions but not the actual values of $z$ and $s_{z}$ as was originally proposed by Ratcliff and Rouder^15^ when introducing the parameter $s_{z}$. The issue of how parameters are fixed between conditions was moot for the starting point of the accumulation (parameter $z$) since it was fixed to half-way between the two boundaries but was critical for the variability of the starting point (parameter $s_{z}$) as discussed in the Results section. On the other hand, DMAT fixes the raw values of the parameters $z$ and $s_{z}$ across conditions. However, one problem that arises is that the parameter $s_{z}$ is constrained so that $z + s_{z} < a$. This is done to ensure that the accumulation always starts between the two boundaries and, indeed, it is arguably nonsensical to assume that the starting point of the accumulation can be beyond either one of the boundaries. However, the “extremely fast” condition in our experiment is naturally fit by a very small value of the boundary $a$. Because the parameter $s_{z}$ is constrained to be the same in all conditions (including the “extremely fast” condition), this led to $s_{z}$ always taking very small values thus minimizing the size of the starting point variability in all conditions. Since this resulted in bad model fits, in one set of simulations, we removed the constraint that $z + s_{z} < a$. We note that while this decision allowed for better model fits (specifically for the difference between error and correct RTs), it creates conceptual difficulties for the diffusion model regarding the plausibility of the accumulation starting beyond the two boundaries.

The second set of model fits allowed all diffusion model parameters to vary freely between conditions, and therefore we refer to them as “free” fits. We fit each combination of Gabor patch contrast and SAT setting independently from all other data in the experiment using six free parameters (for drift rate, boundary, non-decision time, drift rate variability, non-decision time variability, and starting point variability; the starting point was again set halfway between the two boundaries and was not fit as a free parameter). Overall, across the 25 combinations of contrast x SAT setting, we estimated a total of 150 parameters for each subject. However, because performance in the “extremely fast” condition was at chance for many subjects, we only analyzed the parameter fits for the four SAT conditions where performance was above chance for all subjects. We employed repeated measures ANOVAs to test which diffusion model parameters varied as a function of contrast and SAT setting.

All fits were performed using the default features in both HDDM and DMAT. Specifically, the HDDM fits were performed using Bayesian inference, drawing 4000 samples from posterior using Markov Chain Monte Carlo (MCMC) technique and discarding the first 50 samples. To increase the robustness of the estimation, 5% of the data were considered as outliers and modeled using a uniform distribution not generated by a diffusion process^22^ but choosing different values here did not substantially change the results. DMAT has a slight discrepancy between the parameter ranges allowed for the fitting procedure and the procedure for simulating data for a given set of parameters. Specifically, the allowable ranges are larger for two of the parameters during fitting, thus creating situations where DMAT cannot simulate data based on the recovered parameters. To avoid this issue, we altered the allowable ranges during the fitting to be the same as the allowable ranges for data simulation. Specifically, we set the absolute value of the estimated drift rate to lower than 0.5 and its variability to be lower than 0.3. The parameters obtained with these restrictions were typically identical with the ones obtained using the default ranges.

## Supplementary Information

Supplementary Information.

## Data Availability

All raw data and analyses files are freely available at https://osf.io/fw5dc.
