# Supplementary material for: Qualitative speed-accuracy tradeoff effects that cannot be explained by the diffusion model under the selective influence assumption
Source: Sci Rep. 2021 Jan 8;11:45. doi: 10.1038/s41598-020-79765-2 (PMC7794484; doi:10.1038/s41598-020-79765-2)
Supplement: Supplementary file 1 — Supplementary Information. [file 41598_2020_79765_MOESM1_ESM.docx]

**Supplementary Material**

**Article title:** Qualitative speed-accuracy tradeoff effects that cannot be explained by the diffusion model under the selective influence assumption

**Authors**: Farshad Rafiei & Dobromir Rahnev

**Affiliation:** School of Psychology, Georgia Institute of Technology, Atlanta, GA

**Supplementary Figure 1. Individual d’-RT plots**. The plots show, for each subject, the d’ and mean RT for each contrast X SAT condition combination. As can be appreciated, subjects 2, 8, 10, 15, and 18 responded much more slowly and accurately in the “extremely fast” condition than the rest of the subjects. We therefore excluded these five subjects in the main analyses. However, including them did not alter any of our conclusions (**Supplementary Figure 2**).

**Supplementary Figure 2. Results when all 20 subjects are included**. Analyzing all 20 subjects together led to very similar results as in our main analyses where we excluded five subjects for not responding fast enough in the “extremely fast” condition. Specifically, we performed again all of the repeated measures ANOVAs from the main analyses and found that contrast, SAT level, and the interaction between contrast and SAT level were still significant for all four analyses (all *p*’s < .0004) except the main effect of contrast (F(4,76) = 1.39, *p* = 0.2) and contrast X SAT interaction (F(19,66) = 1.9, *p* = 0.8), which were also not significant in the main analyses. More importantly, we found the same patterns for the shapes of the curves as a function of SAT level: when averaged across contrasts, we found no significant quadratic component in the d’-RT curves (t(19) = -1.78, *p* = .09) but significantly U-shaped curves for the difference between error and correct RT (t(19) = 3.49, *p* = 0.002), the $\frac{SD\left( RT \right)}{mean\left( RT \right)}$ ratio (t(19) = 5.89, *p* = 1.1*10^-5^), and the skewness of the RT distribution (t(19) = 4.37, *p* = 0.0003). Further, the pattern of minima and maxima observed remained the same as in our main analyses (Figure 3). Therefore, the pattern of results we observed was not influenced by the exclusion of the five subjects with high RTs in the “extremely fast” condition. All notation is identical to Figure 3 in the main text.

**Supplementary Figure 3. Individual subject plots of the d’-RT relationship for each contrast**. One potential concern with our experimental design is that subjects may treat the “extremely fast” condition as a purely detection task and thus perform it in a qualitatively different fashion. To assess this possibility we plotted the (d’,RT) pairs for each subject, separately for each contrast. As can be seen in the plots above, the different SAT conditions form a single continuum with non-trivial overlap between different conditions and this effect appears for each of the five contrasts. These results suggest that the “extremely fast” condition is unlikely to be qualitatively different from the remaining conditions.

**Supplementary Figure 4. Curves for the ratio between SD and mean RT for different RT exclusion criteria**. Our original analyses excluded all RTs faster than 150 ms and slower than 1,500 ms (these results are reproduced here for comparison purposes in the upper left panel). We repeated the analyses after varying the RT exclusion criteria. Specifically, we used either 150 or 250 ms for the lower threshold and either 1,500 or 2,500 ms for the upper threshold. As can be seen in the panels above, all combinations of RT exclusions led to the same qualitative results such that the $\frac{SD\left( RT \right)}{mean\left( RT \right)}$ ratio curves were robustly U-shaped. Indeed, quadratic fits showed a positive quadratic coefficient when averaged across the five contrasts in all cases with the original exclusion criteria actually producing the highest p value (for included RTs of 150-1,500 ms: t(14) = 4.96, *p* = .0002; for included RTs of 150-2,500 ms: t(14) = 7.13, *p* = 5.1*10^-6^; for included RTs of 250-1,500 ms: t(14) = 6.83, *p* = 8.1*10^-6^; for included RTs of 250-2,500 ms: t(14) = 8.26, *p* = 9.4*10^-7^). Therefore, the robustly U-shaped curves for the $\frac{SD\left( RT \right)}{mean\left( RT \right)}$ ratio as a function of SAT level are not due to the specific RT exclusion criteria that we chose. All notation is identical to Figure 3 in the main text.

**Supplementary Figure 5.** $\frac{\boldsymbol{SD}\left( \boldsymbol{RT} \right)}{\boldsymbol{mean}\left( \boldsymbol{RT} \right)}$ **ratio as a function of mean RT**. In the main text of the paper we described that if the relationship between SD and mean of RT distributions is linear such that $SD(RT)=a+b*mean(RT)$, then the ratio between the SD and mean of the RT distribution ($\frac{SD\left( RT \right)}{mean\left( RT \right)}$) would equal $b+\frac{a}{mean(RT)}$. Therefore, this ratio should be either a monotonically increasing or monotonically decreasing function of mean RT. However, for consistency with the other figures, we investigated the $\frac{SD\left( RT \right)}{mean\left( RT \right)}$ ratio as a function of SAT level rather than directly as a function of mean RT. Therefore, here we plot $\frac{SD\left( RT \right)}{mean\left( RT \right)}$ against mean RT and show that we still obtain robustly U-shaped curves. Indeed, a quadratic fit to the data from each contrast showed positive quadratic coefficient when averaged across the five contrasts (t(14) = 3.64, *p* = .003). In addition, the quadratic coefficient was significantly positive for four of the five individual contrasts (contrast 1: t(14) = 4.28, *p* = .0008; contrast 2: t(14) = 2.83, *p* = .01; contrast 3: t(14) = 3.98, *p* = .001; contrast 4: t(14) = 2.68, *p* = .018; contrast 5: t(14) = 1.96, *p* = .07). All notation is identical to Figure 3 in the main text.

**Supplementary Figure 6. RT standard deviation as a function of mean RT**. In the main text of the paper we plotted $\frac{SD\left( RT \right)}{mean\left( RT \right)}$ as a function of SAT level. In addition, Supplementary Figure 5 showed $\frac{SD\left( RT \right)}{mean\left( RT \right)}$ as a function of mean RT. To give further intuition about the underlying relationship, here we plot the standard deviation of RT, $SD\left( RT \right)$, as a function of mean RT, $mean\left( RT \right)$. The same non-linearity can be seen in the graph too. All notation is identical to Figure 3 in the main text.

**Supplementary Figure 7. Skewness of the RT distributions for different RT exclusion criteria**. Our original analyses excluded all RTs faster than 150 ms and slower than 1,500 ms (these results are reproduced here for comparison purposes in the upper left panel). We repeated the analyses after varying the RT exclusion criteria. Specifically, we used either 150 or 250 ms for the lower threshold and either 1,500 or 2,500 ms for the upper threshold. As can be seen in the panels above, all combinations of RT exclusions led to the same qualitative results such that the skewness curves were robustly U-shaped. Indeed, quadratic fits showed a positive quadratic coefficient when averaged across the five contrasts in all cases (for included RTs of 150-1,500 ms: t(14) = 4.89, *p* = .0002; for included RTs of 150-2,500 ms: t(14) = 5.51, *p* = 7.7*10^-5^; for included RTs of 250-1,500 ms: t(14) = 4.23, *p* = .0008; for included RTs of 250-2,500 ms: t(14) = 4.87, *p* = .0002). Therefore, the robustly U-shaped curves for the skewness of the RT distribution as a function of SAT level are not due to the specific RT exclusion criteria that we chose. All notation is identical to Figure 3 in the main text.


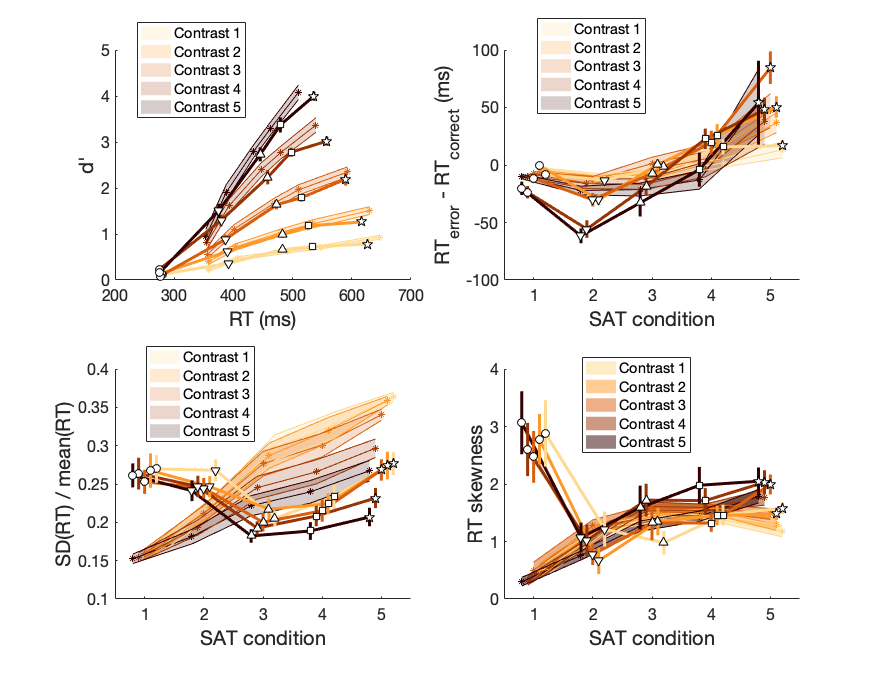


**Supplementary Figure 8. DMAT fits with custom settings that allow the starting point to be outside of the boundaries**. We fit the diffusion model to the data using the software package DMAT with custom settings that allowed the parameter $s_{z}$ to be larger than $\frac{a}{2}$ (thus allowing the process of accumulation to start beyond the boundaries and thus terminate immediately, which could be seen as non-sensical). The results were very similar to the default DMAT fits except that the RT difference between error and correct trials could now take negative values (upper right panel) and therefore qualitatively replicate the U-shaped pattern observed in the data. However, even then, the fits were far from perfect, especially for the “fast” condition where the large differences between $RT_{error}$ and $RT_{correct}$ were not reproduced. All notation is identical to Figure 4 in the main text.


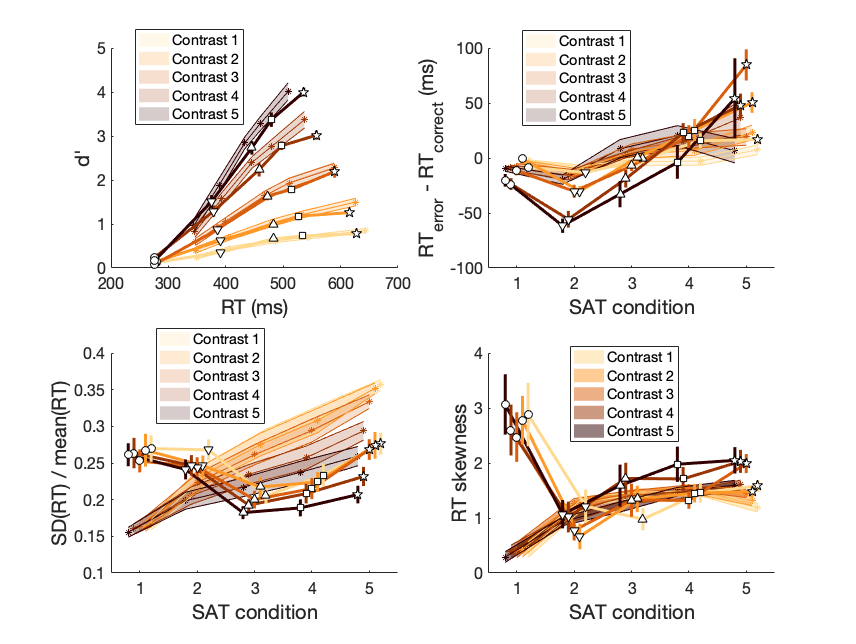


**Supplementary Figure 9. Diffusion model fits where both the boundary** $\boldsymbol{a}$ **and the starting point** $\boldsymbol{s}_{\boldsymbol{z}}$ **can vary across SAT conditions**. We fit the diffusion model to the data using the software package DMAT by allowing both the boundary $a$ and the starting point $s_{z}$ to vary across SAT conditions (only the drift rate was allowed to vary across different contrast levels). This resulted in a total of 18 free parameters. The results were overall very similar to both default (**Figure 5**) and custom (**Supplementary Figure 8**) DMAT fits where $s_{z}$ did not vary across conditions. Just as with the custom fits, the model could produce negative numbers for the RT difference between error and correct trials and therefore qualitatively replicated the U-shaped pattern observed in the data. However, the fits were again far from perfect, especially for the “fast” condition where the large differences between $RT_{error}$ and $RT_{correct}$ were not reproduced. All notation is identical to Figure 4 in the main text.


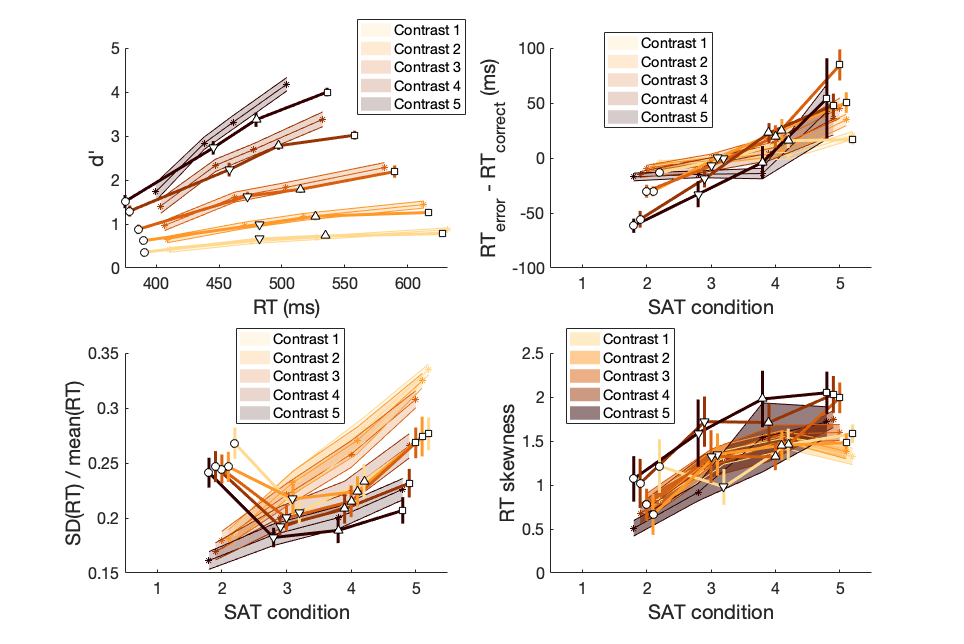


**Supplementary Figure 10. Diffusion model fits when the “extremely fast” condition is excluded**. We investigated whether the failures of the diffusion model to fit the data stemmed from the “extremely fast” condition. We used DMAT to fit all data but excluded the data from the “extremely fast” condition. The results were very similar to the ones obtained in the previous fits (if the “extremely fast” condition is to be ignored). Specifically, the model still could not account for the RT difference between error and correct trials in the “fast” condition or the U-shaped curve for the ratio between the standard deviation and mean of the RT distribution, but could now account well for the RT skewness (which is also true in Figure 4 and 5 when the “extremely fast” condition is ignored). All notation is identical to Figure 4 in the main text.

**Supplementary Figure 11. Additional model simulations**. Figures 6 and 7 in the main paper simulated the predictions of the diffusion model for different values of the drift rate variability ($\eta$) or starting point variability ($s_{z}$). Here, we show additional simulations where these parameters take different values, and additionally show the predictions of the diffusion model when the parameter drift rate ($v$) takes values of .05 and .25 (the drift rate was fixed to .15 in Figures 6 and 7). As can be seen, the predictions of the diffusion model remain the same as discussed in the main paper for all parameter combinations.


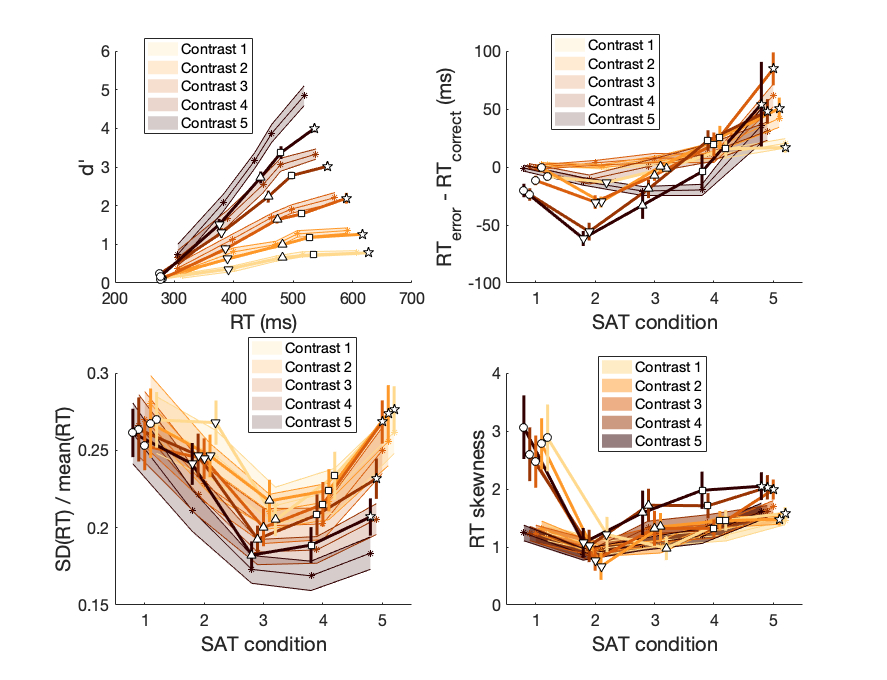


**Supplementary Figure 12. Diffusion model fits when each condition is modeled independently**. We used HDDM (DMAT led to very similar results) and fit each of the 25 conditions in the experiment (5 contrast levels X 5 SAT levels) independently from the rest. This resulted in 150 total free parameters. Despite the extreme flexibility afforded by this type of fit, the diffusion model still could not account for several features of the data. Most notably, it could not fit the large difference between error and correct RTs for the “fast” condition, as well as the U-shaped pattern for the RT skewness. On the other hand, the model was now able to fit the $\frac{SD\left( RT \right)}{mean\left( RT \right)}$ curve. All notation is identical to Figure 4 in the main text.

**Supplementary Figure 13. Dependence of each diffusion model parameter on contrast and SAT when DMAT is used for model fitting**. To test the “selective influence” assumption of the diffusion model, we fit each combination of contrast and SAT level (except for the extremely fast SAT condition) with the diffusion model independently from all other conditions. In Figure 8, we reported the results of these analyses when implemented using HDDM. Here we report the results of the same analyses but implemented using DMAT. We again observed a significant effect of both contrast and SAT on every single one of the six diffusion model parameters. Specifically, we found significant effects of contrast on the drift rate (F(4,56) = 412.41, *p* = 8.03*10^-41^), boundary (F(4,56) = 36.9, *p* = 4.3*10^-15^), non-decision time (F(4,56) = 30.98, *p* = 1.3*10^-13^), drift rate variability (F(4,56) = 9.62, *p* = 5.4*10^-6^), non-decision time variability (F(4,56) = 46.26, *p* = 4.01*10^-17^), absolute starting point variability (F(4,56) = 3.22, *p* = .019), and relative starting point variability (F(4,56) = 3.199, *p* = .0195). Similarly, we found significant effects of SAT on the drift rate (F(3,42) = 28.39, *p* = 3.4*10^-10^), boundary (F(3,42) = 65.96, *p* = 6.2*10^-16^), non-decision time (F(3,42) = 24.89, *p* = 2.1*10^-9^), drift rate variability (F(3,42) = 13.91, *p* = 1.9*10^-6^), non-decision time variability (F(3,42) = 6.34, *p* = 0.001), absolute starting point variability (F(3,42) = 7.96, *p* = .0003), and relative starting point variability (F(3,42) = 45.12, *p* = 3.4*10^-13^). Finally, and again contrary to the diffusion model predictions, there was a significant interaction between contrast and SAT for all six parameters (drift rate: F(14,30.6) = 3.27, *p* = .0002, boundary: F(14,41.6) = 4.29, *p* = 8.5*10^-12^, non-decision time: F(14,48.3) = 4.25, *p* = 3.9*10^-15^, drift rate variability: F(14,30.9) = 1.98, *p* = 0.0003, non-decision time variability: F(14,46.3) = 2.06, *p* = 0.0008, absolute starting point variability: F(14,37.8) = 3.1, *p* = 1.7*10^-6^, and relative starting point variability: F(14,40.3) = 1.78, *p* = 1.4*10^-6^). Just like the results from HDDM, these results obtained using DMAT strongly question the “selective influence” assumption of the diffusion model. All notation is identical to Figure 8.

**
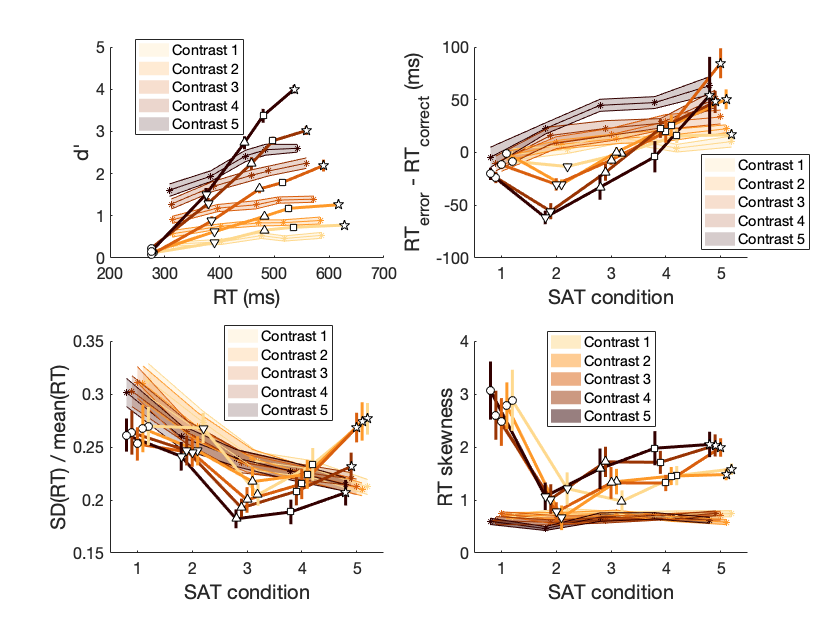
**

**Supplementary Figure 14. LBA fits**. In addition to the fits with the diffusion model, we fit the linear ballistic accumulation (LBA) model to the data. Notably, LBA provided worse fits than the diffusion model. This is especially obvious in the case of the d’-RT functions where LBA was unable to reproduce the steep increases in both d’ and RT observed with increasing stress on accuracy. In addition, LBA could not reproduce any of the three U-shaped curves. LBA parameter recovery was performed using Dynamic Models of Choice (DMC) toolbox in R, which is based on the response time distributions (rdists 0.11-2) package. For each subject, we estimated starting point (A), boundary (B = b - A), drift rate (v), and non-decision time (t0). Based on the selective influence assumption, the boundary parameter varied with SAT condition, while the drift rate varied with contrast; the remaining parameters were held constant across conditions. Therefore, a total of 12 parameters were estimated for each subject. The basis of the parameter recovery in DMC is MCMC sampling and we ran 1500 iterations for each MCMC chain. All notation is identical to Figure 4.

**Supplementary Figure 15. Additional LBA fits.** We extended the flexibility of the LBA model fit in Supplementary Figure 14 by allowing the drift rates for both the correct and error choices to vary with contrast. Thus, for each subject we estimated starting point (A), boundary (B = b - A), drift rate for correct (v_correct_) and error (v_error_) choices, variability in drift rates (sv_correct_ and sv_error_) and non-decision time (t0). Based on the selective influence assumption, the boundary parameter varied with SAT condition, whereas the drift rates for correct and error choices both varied with contrast. The remaining parameters were held constant across conditions. Therefore, a total of 19 parameters were estimated for each subject. Model fitting was performed as in Supplementary Figure 14. As can be seen from the figure, the additional flexibility afforded by allowing the drift rates for correct and error choices to both vary with contrast did not meaningfully improve the fits. All notation is identical to Figure 4.
